# A multicenter study on Leigh syndrome: disease course and predictors of survival

**DOI:** 10.1186/1750-1172-9-52

**Published:** 2014-04-15

**Authors:** Kalliopi Sofou, Irenaeus F M De Coo, Pirjo Isohanni, Elsebet Ostergaard, Karin Naess, Linda De Meirleir, Charalampos Tzoulis, Johanna Uusimaa, Isabell B De Angst, Tuula Lönnqvist, Helena Pihko, Katariina Mankinen, Laurence A Bindoff, Már Tulinius, Niklas Darin

**Affiliations:** 1 Department of Paediatrics, University of Gothenburg, The Queen Silvia’s Children Hospital, SE-416 85 Gothenburg, Sweden; 2 Department of Neurology, Erasmus MC, Rotterdam, The Netherlands; 3 Department of Paediatric Neurology, Children’s Hospital, Helsinki University Central Hospital, University of Helsinki, Helsinki, Finland; 4 Research Programs Unit, Molecular Neurology, Biomedicum-Helsinki, University of Helsinki, Helsinki, Finland; 5 Department of Clinical Genetics, Copenhagen University Hospital Rigshospitalet, Copenhagen, Denmark; 6 Centre for Inherited Metabolic Diseases, Karolinska University Hospital, Stockholm, Sweden; 7 Department of Paediatric Neurology, University Hospital Vrije Universiteit Brussel (VUB), Brussels, Belgium; 8 Department of Neurology, Haukeland University Hospital, Bergen, Norway; 9 Department of Clinical Medicine, University of Bergen, Bergen, Norway; 10 Institute of Clinical Medicine/Department of Paediatrics, University of Oulu, Oulu, Finland; 11 Medical Research Center, Oulu University Hospital, University of Oulu, Oulu, Finland; 12 Länsi-Pohja Central Hospital, Kemi, Finland

**Keywords:** Leigh syndrome, Natural history, Survival, Relapse, Prognosis, Diagnostic criteria

## Abstract

**Background:**

Leigh syndrome is a progressive neurodegenerative disorder, associated with primary or secondary dysfunction of the mitochondrial oxidative phosphorylation. Despite the fact that Leigh syndrome is the most common phenotype of mitochondrial disorders in children, longitudinal natural history data is missing. This study was undertaken to assess the phenotypic and genotypic spectrum of patients with Leigh syndrome, characterise the clinical course and identify predictors of survival in a large cohort of patients.

**Methods:**

This is a retrospective study of patients with Leigh syndrome that have been followed at eight centers specialising in mitochondrial diseases in Europe; Gothenburg, Rotterdam, Helsinki, Copenhagen, Stockholm, Brussels, Bergen and Oulu.

**Results:**

A total of 130 patients were included (78 males; 52 females), of whom 77 patients had identified pathogenic mutations. The median age of disease onset was 7 months, with 80.8% of patients presenting by the age of 2 years. The most common clinical features were abnormal motor findings, followed by abnormal ocular findings. Epileptic seizures were reported in 40% of patients. Approximately 44% of patients experienced acute exacerbations requiring hospitalisation during the previous year, mainly due to infections. The presence of pathological signs at birth and a history of epileptic seizures were associated with higher occurrence of acute exacerbations and/or relapses. Increased lactate in the cerebrospinal fluid was significantly correlated to a more severe disease course, characterised by early onset before 6 months of age, acute exacerbations and/or relapses, as well as brainstem involvement. 39% of patients had died by the age of 21 years, at a median age of 2.4 years. Disease onset before 6 months of age, failure to thrive, brainstem lesions on neuroimaging and intensive care treatment were significantly associated with poorer survival.

**Conclusions:**

This is a multicenter study performed in a large cohort of patients with Leigh syndrome. Our data help define the natural history of Leigh syndrome and identify novel predictors of disease severity and long-term prognosis.

## Introduction

Leigh syndrome or subacute necrotizing encephalomyelopathy, is a genetically heterogeneous, progressive neurodegenerative disorder, that is usually associated with defects involving mitochondrial oxidative phosphorylation (OXPHOS). This disorder primarily affects infants and young children and is considered to be the most common, distinct phenotype among OXPHOS disorders in children with an estimated pre-school incidence in western Sweden of 1 per 34,000 births [1].

Leigh syndrome was first described as a distinct neuropathological entity with focal, bilaterally symmetrical, subacute necrotic lesions extending from the thalamus to the brainstem and the posterior columns of the spinal cord [2]. Since then, the diagnosis of Leigh syndrome has been based on symmetrical lesions in one or more areas of the central nervous system, including the basal ganglia, diencephalon, brainstem, cerebellum and spinal cord, on either post mortem examination or on neuroimaging [3,4]. Typically, the affected areas appear hypodense on computed tomography (CT) and show hyperintense signal on T2-weighted and hypointense signal on T1-weighted magnetic resonance imaging (MRI) [5,6].

Onset of disease occurs typically between 3 and 12 months of age, with disease progression and death within 2 years. Later onset and slower progression have also been reported [3,4,7-9]. Clinical manifestations include motor delay, mental retardation and/or progressive cognitive decline, hypotonia, dyskinesia, akinesia, ataxia, dystonia and brainstem dysfunction, including respiratory abnormalities, swallowing dysfunction, ophthalmological manifestations and abnormal thermoregulation [3,10].

Leigh syndrome can be inherited as a mitochondrial trait, as an autosomal recessive trait due to mutations in nuclear genes encoding mitochondrial respiratory chain complex subunits or complex assembly proteins [11] and X-linked related to defects in pyruvate dehydrogenase complex (PDHc) due to mutations in the *PDHA1* gene (MIM# 300502) [12].

Leigh syndrome is a rare and heterogeneous disease making it difficult to obtain large cohorts of patients to study. Although the clinical features of Leigh syndrome have been described in published reviews, studies of longitudinal data evaluating the natural history of the disease in a large cohort of patients are lacking. Furthermore, the clinical reports published so far have been based on small numbers of patients and could not assess predictors of survival. The Mitochondrial Clinical and Research Network (MCRN) was established to facilitate research collaboration among centers specialising in mitochondrial diseases. This project, which aims to analyse the phenotypic and genotypic spectrum of patients with Leigh syndrome, characterise the clinical course and identify predictors of survival in a large cohort of patients is the first joint study of this network.

## Materials and methods

### Study design and population

The study was conducted as a retrospective analysis in eight European centers specialising in mitochondrial diseases that constitute the MCRN; Gothenburg, Rotterdam, Helsinki, Copenhagen, Stockholm, Brussels, Bergen and Oulu. We included patients with Leigh syndrome that were diagnosed and followed at the participating centers and that fulfilled both of the following inclusion criteria: (i) clinical features compatible with Leigh syndrome, such as psychomotor regression, dystonia, ataxia and/or brainstem dysfunction and (ii) MRI or CT or neuropathological findings of Leigh syndrome, as follows: bilateral symmetrical lesions in the basal ganglia, and/or thalamus, and/or brainstem. Patients were excluded from the study if they had known syndromic mitochondrial phenotypes other than Leigh syndrome.

Patient data were collected with the help of an electronic-Case Report Form (e-CRF) that was completed by one designated investigator at each center (Additional file 1: Table S1). Patient data included medical history and survival status/date of last follow-up, family history of neurological disorders, biochemical findings, respiratory chain enzyme activities, muscle histology and histochemistry, liver histology, brain neuroimaging, genetic findings and type of medical treatment for Leigh syndrome. Preterm birth was defined as childbirth occurring at less than 37 completed weeks of gestation. The term ‘pathological signs at birth’ was defined as any abnormal clinical and/or biochemical findings at birth. Resistance to antiepileptic treatment was defined as per the ILAE (International League Against Epilepsy) definition [13]. A detailed list with the definitions of the terms used in this study was provided as an attachment in the e-CRF (Additional file 1: Table S1).

Each participating center received approval by the local ethical committee Ethical Review Board at the University of Gothenburg, as per the standing local regulatory requirements, prior to the initiation of the study. Interim monitoring procedures ensured the validity and integrity of the collected data.

### Statistical analysis

Statistical analysis was performed using the statistical software package SAS v9.3. Due to the rarity of the disease, no sample size calculation was performed in advance and the study was not powered for specific statistical hypotheses. The statistical evaluations performed were therefore mainly exploratory.

Kaplan-Meier analysis was used to estimate survival outcomes. Differences in survival between groups were evaluated with the Log rank test. Cox regression analysis was performed to assess potential predictors of survival. Wilcoxon-Mann–Whitney test was applied for the evaluation of differences in continuous variables between groups. Chi-square test and Fisher’s exact test were used to test the association between categorical variables. Multiple logistic regression analysis was performed to further investigate the relationship between binary response variables and potential predictors of survival. Backward selection method was used to identify the best fitting model. All statistical tests were two-sided and performed at a 0.05 significance level.

## Results

### Demographics and family history

A total of 130 patients with Leigh syndrome were enrolled into the study, among whom 11 sibling pairs. There was a slight male preponderance (78 males; 52 females). The majority of the patients were European (73.1%), followed by North-Africans (9.2%), Turks (3.8%), Kurds (2.3%) and other ethnic groups (11.6%).

Parental consanguinity was present in 31 patients from 24 families. These were European (n = 7), North-African (n = 7), Turkish (n = 4), Kurdish (n = 2), Pakistani (n = 2), Lebanese (n = 1) and Iraqi (n = 1). The presence of mitochondrial disorders that did not fulfill the criteria of Leigh syndrome was reported in siblings to three of our patients with Leigh syndrome. Two of them, one with neonatal encephalopathy and another with infantile cardiomyopathy died early and autopsy was not performed. The third refers to a sibling with clinical features and course similar to his brother with Leigh syndrome, but without the neuroimaging findings described in our inclusion criterion (ii).

Abnormal neurological findings were reported in three mothers to children with Leigh syndrome, who carried the same mutations as their children -two with PDHc deficiency and one with a pathogenic mutation in *MT-ATP6*. One of these mothers suffered from polyneuropathy and ataxia, one mother had intentional tremor, hyperreflexia of lower extremities, pes cavus and difficulty in walking on heels, and the third mother had both intentional and resting tremor.

### Perinatal history

19 patients (14.6%) were born preterm. Most pregnancies were uneventful (77.7%); the most common causes of complicated pregnancy were preeclampsia and oligohydramnios. 13 patients (10.0%) were born small for gestational age, while two patients presented intrauterine growth restriction. Microcephaly was evident at birth in six patients (4.6%). The median Apgar score at 1, 5 and 10 minutes was 9-9-10 (Q25: 7-9-9; Q75: 9-10-10). Nine patients (6.9%) had respiratory difficulties at birth, of whom eight required the following interventions: intubation/ventilation (n = 4), oxygen by mask (n = 2) and nasal CPAP (n = 2). 30 patients (23.1%) were reported to have had pathological signs at birth, which apart from respiratory difficulties, were hypotonia/floppiness (n = 7); cardiac complications (n = 5); lactic acidosis (n = 4); feeding/sucking difficulties (n = 3); dysmaturity (n = 2); hypoglycaemia (n = 2); hyperbilirubinaemia (n = 2); hyperammonaemia (n = 2); epileptic seizures (n = 1); hypertonia (n = 1); contractures (n = 1), dysmorphic features (n = 1) and increased plasma lactate with hyponatraemia (n = 1).

### Disease onset and age at diagnostic testing

**Figure 1:**
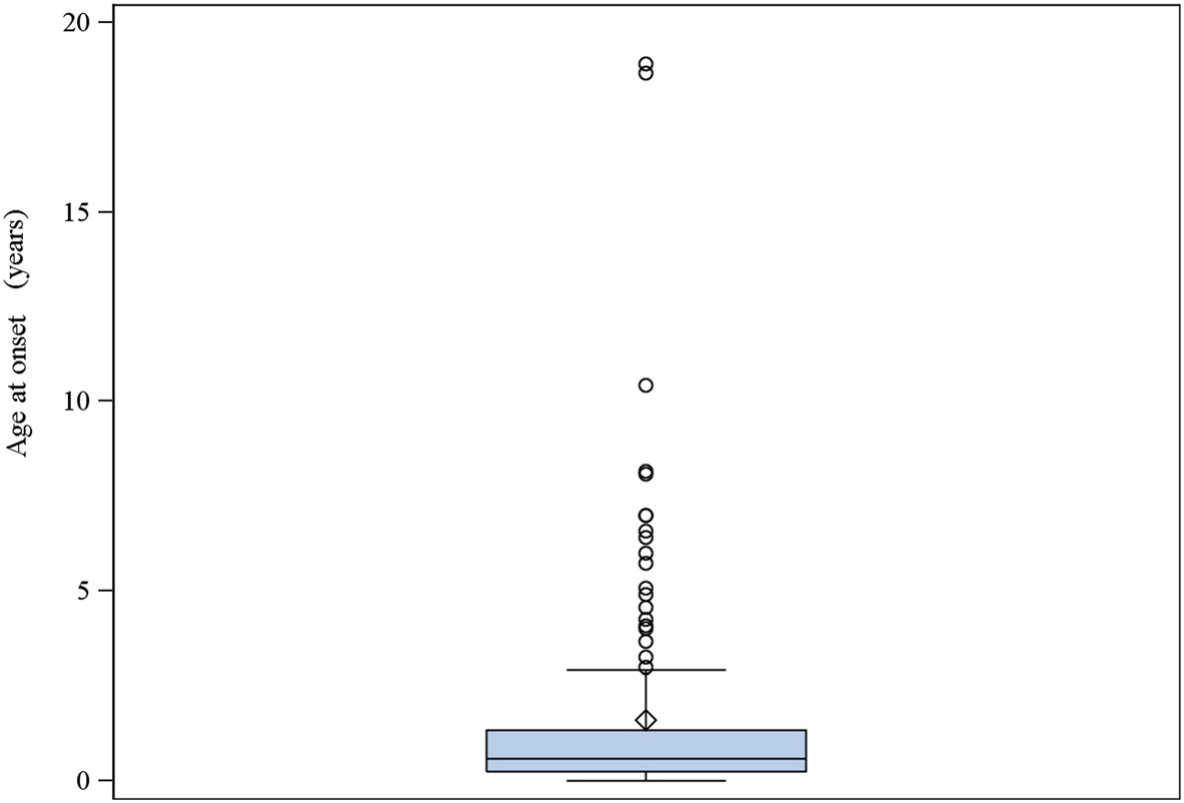
Median age of disease onset.

The median age of disease onset was 7 months, with 80.8% presenting by the age of 2 years (Figure 1). No difference between genders was observed regarding age of onset (*p* = 0.775). Perinatal onset of disease was reported in 17 patients (13.1%), while three patients were reported to have intrauterine onset. One of these patients had cardiomegaly and enlarged ventricles and germinolytic cysts on prenatal ultrasound, as well as hypotonia and lactic acidosis at birth; genetic etiology in this patient is still unknown. The second patient with reported intrauterine onset presented with contractures at birth, had an Apgar score of 2-5-8 and respiratory complications necessitating intubation. This patient was found to have *SUCLA2* mutations. The third patient had *MT-ND3*-associated Leigh syndrome and an unknown perinatal history.

The patients with the latest age of onset -above the 90^th^ percentile of the age-of-onset distribution of all included patients- were 13 patients with age of onset above 4.9 years. Their clinical presentations and disease course are summarised in Table 1.

**Table 1:** Clinical presentation and disease course for the 13 patients with the latest disease onset (above 4.9 years)

ID*	**Age of onset**	**Clinical features at onset**	**Exacerbations/relapses requiring hospitalisation**	**Survival status**	**Genetic etiology**
**84**	5 y 1 m	Hypotonia, mental retardation	No	Alive at 7 y 6 m	Unknown
**66**	4 y 11 m	Dystonia, spasticity, hyperreflexia	No	Alive at 33 y	*C20orf7*
**94**	8 y1 m	Dystonia, hypertonia	No	Alive at 18 y 10 m	Unknown
**95**	18 y 8 m	Gastrointestinal symptoms	Yes	Died at 19 y 6 m	Unknown
**15**	6 y 7 m	Hypotonia, ophthalmoplegia, fatigue	No	Alive at 14 y 7 m	*MT-ATP6*
**99**	5 y 9 m	Hypotonia, pos Babinski sign, ophthalmoplegia	Yes	Alive at 33 y	Unknown
**103**	6 y 5 m	Dystonia, hypertonia, pos Babinski sign	No	Alive at 8 y 6 m	Unknown
**114**	7 y	Dystonia	No	Lost to follow-up at 10 y	Unknown
**118**	7 y	Gastrointestinal symptoms	No	Alive at 12 y 5 m	Unknown
**5**	6 y	Discoordination	No	Alive at 19 y 8 m	*MT-ND3*
**124**	10 y 5 m	Hypo-hypertonia, dystonia, hypokinesia, dysarthria, hyperreflexia, sucking dysfunction, bradyphrenia	Yes	Alive at 20 y 8 m	Unknown
**127**	8 y 2 m	Hypertonia, hyperreflexia	No	Alive at 14 y	Unknown
**25**	19 y	Spasticity, hemiparesis	No	Alive at 58 y	*MT-ATP6*

*The ID numbers correspond to the ID numbers of the respective patients on Table 4.

*Y* years; *m* months.

The patients underwent diagnostic testing for suspected mitochondrial disease at a median age of 2.3 years (interquartile range Q25-Q75: 0.8-6.3 years). The median elapsed time from disease onset to diagnostic testing was 0.9 years (interquartile range Q25-Q75: 0.2-3.1 years).

### Clinical features at onset

**Figure 2:**
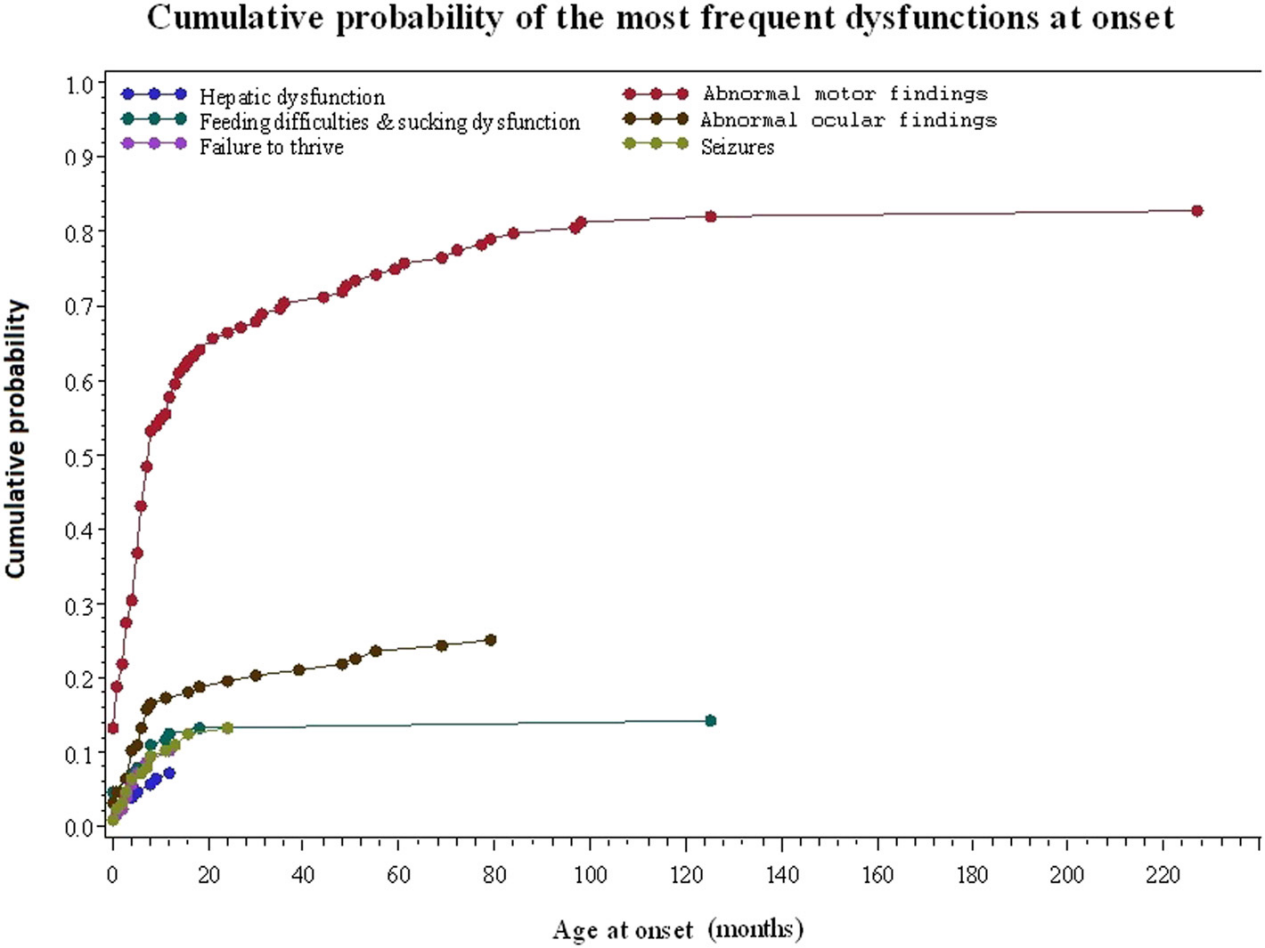
Overview of clinical features at onset.

Leigh syndrome presented initially with abnormal motor findings in the vast majority of patients (82.8%). Other common features were abnormal ocular findings (25.0%), feeding/sucking difficulties (14.1%), epileptic seizures (13.3%) and failure to thrive (10.2%). The clinical features at onset in relation to age at onset are depicted in Figure 2.

### Clinical features throughout the disease course

An overview of the patients’ clinical features is shown in Table 2. The most common clinical features were abnormal motor findings (99.2%), followed by abnormal ocular findings (60.8%) (Table 2). More than half of the patients had at least three affected organ systems during the course of the disease. The three most frequently involved systems were the motor, visual and gastrointestinal system (Table 2).

**Table 2:** Overview of clinical features throughout the disease course

**Frequency of affected organs/systems (n = 130)**	**N**	**%**
Abnormal motor findings	129	99.2
Abnormal ocular findings	79	60.8
Feeding difficulties	59	45.4
Epileptic seizures	51	39.2
Respiratory dysfunction	49	37.7
Mental retardation	48	36.9
Sucking dysfunction	32	24.6
Hearing dysfunction	25	19.2
Cardiac dysfunction	23	17.7
Failure to thrive	21	16.2
Hepatic dysfunction	16	12.3
Microcephaly	15	11.5
Peripheral neuropathy	9	6.9
Renal dysfunction	7	5.4
Haematological dysfunction	2	1.5

### Abnormal motor findings

**Figure 3:**
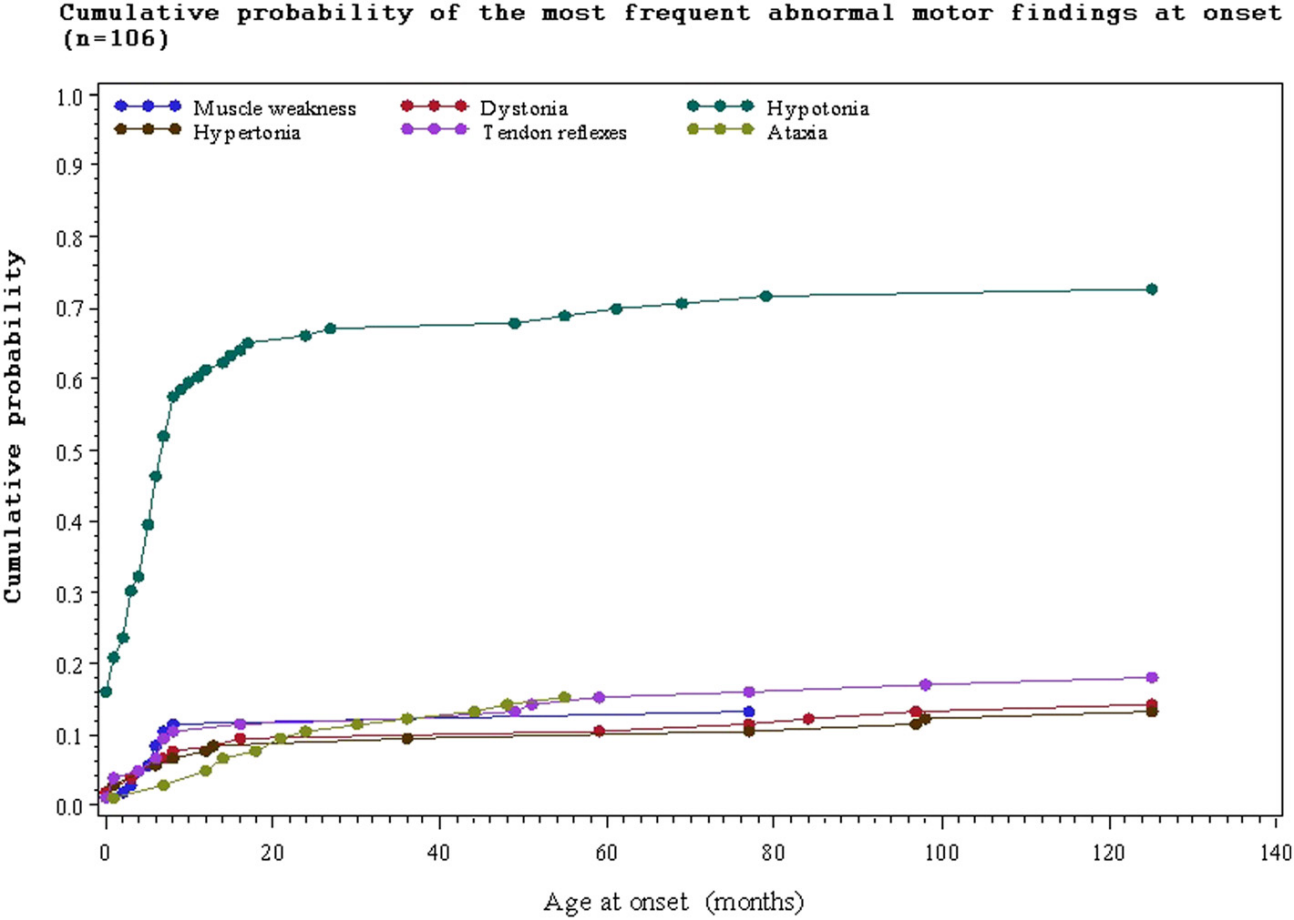
Abnormal motor findings at onset in relation to age of onset.

Hypotonia was the most common motor finding (74.6%), followed by abnormal tendon reflexes (47.7%) and dystonia (44.6%) (Table 3). The most common presenting motor features were hypotonia seen in 59.2%, abnormal tendon reflexes (14.6%) and ataxia (12.3%) (Additional file 2: Table S2). The relationship between motor findings at onset and age at onset of Leigh syndrome are shown in Figure 3. Dystonia, spasticity, hypertonia and choreoathetosis were less frequent at onset but developed later in the disease course (Additional file 2: Table S2).

**Table 3:** Abnormal motor findings

**Type of abnormal motor findings (n = 130)**	**N**	**%**
Hypotonia	97	74.6
Abnormal tendon reflexes§	62	47.7
Dystonia	58	44.6
Babinski sign	55	42.3
Spasticity	47	36.2
Ataxia	45	34.6
Hypertonia	43	33.1
Muscle weakness	34	26.2
Other dyskinetic disorder^	27	20.8
Paresis/palsy	26	20.0
Chorea/athetosis	25	19.2
Cavus feet	10	7.7
Myoclonus	9	6.9
Hypokinesia/bradykinesia	5	3.8

§Decreased or absent (n = 20) vs increased or clonic (n = 42).

^Tremor (n = 7); dysarthria (n = 4); dyscoordination (n = 3); cogwheel phenomenon (n = 3); stereotypies (n = 3); opisthotonus (n = 2); hyperkinesia (n = 2); dyspraxia (n = 1); jitteriness (n = 1); sleeping through phenomenon (n = 1).

### Abnormal ocular findings

Abnormal ocular findings were present in 79 patients (60.8%), the most prevalent being nystagmus (23.8%), followed by strabismus (19.2%), visual impairment (16.2%), optic atrophy (14.6%), ptosis (13.1%) and ophthalmoplegia (12.3%).

### Epileptic seizures

Epileptic seizures were reported in 51 patients (39.2%) and were classified according to ILAE as follows: generalized seizures (22.3%), focal seizures (14.6%) and epileptic spasms (6.1%). In the subgroup of patients with generalized seizures, myoclonic seizures were reported in eight patients and absence epilepsy in three patients. Resistance to antiepileptic treatment was reported in 16 patients.

### Respiratory dysfunction

Respiratory dysfunction was present in 37.7% with hyperventilation and/or abnormal breathing pattern being the most prevalent type (20.0%), followed by apnoea (16.1%), obstructive or restrictive respiratory disease (13.8%) and central hypoventilation (10.0%).

### Cardiac dysfunction

Cardiac dysfunction was present in 23 patients (17.7%), with more than half having hypertrophic cardiomyopathy (9.2%). Arrhythmia/conduction defects were reported in five patients and dilated cardiomyopathy was reported in two patients.

### Other clinical features

59 patients manifested with feeding difficulties (45.4%) sufficient to necessitate tube feeding (20.0%) and/or gastrostomy (33.0%). Mental retardation was found in 48 patients (36.9%). The severity of mental retardation was classified as mild in 11 patients, moderate in 17, severe in 15, profound in three and unspecified in two patients. Hearing impairment was identified in 25 patients and was sensorineural in 22, conductive in two and mixed in one patient. Hepatic dysfunction was present in 16 patients (12.3%), with elevated liver transaminases in 12; structural abnormalities defined by ultrasound or biopsy, including liver steatosis and/or fibrosis in four; severe liver failure in two and hepatomegaly in two patients. Microcephaly was present in 15 patients (11.5%).

Other reported findings in descending order of frequency were: gastro-intestinal dysfunction - including constipation, diarrhea, dysphagia, vomiting, gastritis, megacolon, intestinal paralysis (11.5%); dysarthria (11.5%); hyperhidrosis/excessive sweating (9.2%); motor delay (9.2%); peripheral neuropathy (6.9%); scoliosis (6.9%); speech delay (6.1%); renal dysfunction (5.4%); sleep disturbances (2.3%); hypothermia/hyperthermia (2.3%); psychotic disorders (1.5%) and haematological dysfunction in two patients, one of whom had b-thalassaemia minor and another developed anaemia and thrombocytopenia later in the disease course.

### Biochemical, histological and genetic findings

The main biochemical, histological and genetic findings are summarized in Table 4. 25% of the patients with available lactate values had a maximum lactate in blood and/or cerebrospinal fluid (CSF) below or equal to 2.4 mmol/l, which were considered to be normal. Of those patients with normal lactate levels throughout the disease course, 10 patients had genetically verified disease (Table 4). Elevated lactate in CSF (>2.4 mmol/l) was associated with early onset of Leigh syndrome before 6 months of age (*p* = 0.013), presence of hypotonia (*p* = 0.002), acute exacerbations and/or relapses (*p* = 0.014), brainstem lesions on neuroimaging (*p* = 0.002) and absence of dystonia (*p* = 0.011). No correlations were found between lactate levels in CSF and a history of seizures, abnormal clinical findings other than hypotonia, specific neuroimaging findings or the survival outcome.

**Table 4:** Lactate levels, respiratory chain enzyme activities, presence of muscle pathology and genetic findings in 130 patients with Leigh syndrome

**ID**	**Age of onset**	**B-lactate**	**CSF-lactate**	**Complex deficiency**	**Muscle pathology**	**Genetic etiology**	**ID**	**Age of onset**	**B-lactate**	**CSF-lactate**	**Complex deficiency**	**Muscle pathology**	**Genetic etiology**
**1**	3 m	4.0	4.7	CI	-	*MT -ND1*	**66**	4 y 11 m	1.9	NA	CI	+	*C20orf7*
**2**	2 y 6 m	3.0	3.9	CI	+	*MT -ND1*	**67**	4 y 1 m	4.6	NA	CI	+	*C20orf7*
**3**	IU	2.6	2.5	CI	+§	*MT-ND3*	**68**	1 y	NA	NA	CIV	-	*C12orf65*
**4**	4 m	8.0	6.4	CI	-	*MT-ND3*	**69**	Perinatal	10.2	NA	CI, CII	+	*NDUFA9*
**5**	6 y	1.5	2.5	-	-	*MT -ND3*	**70**	2 m	4.9	2.4	CI	+	*NDUFA12*
**6**	1 y	7.7	NA	CI, CIV	-	*MT -ND3-4*	**71**	3 m	5.7	NA	CI, CIII	-	*NDUFAF2*
**7**	11 m	10.4	2.2	CI, CV	+	*MT -ND5*	**72**	4 m	NA	NA	CI	-	*NDUFAF2*
**8**	Perinatal	22.9	NA	CI, CIII, CIV	+	*MT-ND5*	**73**	6 m	4.1	NA	CI	+	*NDUFV2*
**9**	8 m	1.6	3.7	CI	-	*MT -ND5*	**74**	5 m	15.0	NA	CI	-	*NDUFV2*
**10**	1 y 4 m	5.0	3.2	CI	+	*MT-ND6*	**75**	3 y 3 m	1.6	NA	CI, CIII	+	*NDUFS7*
**11**	1 y 9 m	3.2	4.6	-	+	*MT-ATP6*	**76**	3 m	10.2	4.7	CI	-	*NDFUS4*
**12**	11 m	2.1	NA	NA	NA	*MT-ATP6*	**77**	9 m	3.6	2.0	CIII	-	*BCS1L*
**13**	5 m	4.4	NA	NA	NA	*MT-ATP6*	**78**	7 m	4.2	4.0	CI	+	Unknown
**14**	Perinatal	8.5	5.9	CV	+	*MT-ATP6*	**79**	7 m	1.5	1.5	CII	-	Unknown
**15**	6 y 7 m	1.5	2.4	CV	+	*MT-ATP6*	**80**	6 m	7.0	3.2	CI	+	Unknown
**16**	1 y 2 m	2.1	2.7	CI-IV	-	*MT-ATP6*	**81**	2 y	1.0	1.9	-	-	Unknown
**17**	4 m	4.7	5.2	CI-IV	+	*MT-ATP6*	**82**	1 y 3 m	4.4	NA	-	+	Unknown
**18**	2 y 3 m	1.5	2.7	-	+§	*MT-ATP6*	**83**	Perinatal	2.8	2.0	CIV	+	Unknown
**19**	6 m	3.1	3.3	-	-	*MT-ATP6*	**84**	5 y 1 m	1.1	6.1	NA	NA	Unknown
**20**	4 m	2.4	3.3	-	-	*MT-ATP6*	**85**	1 y 5 m	2.7	2.3	-	+	Unknown
**21**	5 m	5.4	5.7	CI-IV	-	*MT-ATP6*	**86**	1 y 4 m	1.2	NA	-	+	Unknown
**22**	6 m	6.2	6.2	CI, CIII, CIV, CV	-	*MT-ATP6*	**87**	Perinatal	7.5	12.9	CI, CIII	+	Unknown
**23**	3 m	5.5	5.8	CV	-	*MT-ATP6*	**88**	1 y 2 m	5.5	3.0	CI, CIV	+	Unknown
**24**	3 m	6.4	8.1	-	+	*MT-ATP6*	**89**	7 m	4.4	5.9	CI, CII	+	Unknown
**25**	19 y	1.2	NA	NA	NA	*MT-ATP6*	**90**	2 m	5.6	2.1	CI-III	+	Unknown
**26**	1 y 6 m	0.8	2.1	CV	+	*MT-ATP6*	**91**	Perinatal	17.0	NA	-	-	Unknown
**27**	8 m	8.9	2.8	CI, CIV	+	*MT-TW*	**92**	Perinatal	6.7	3.7	CI	-	Unknown
**28**	2 y	14.8	4.0	CI, CIV	+	*MT-TL1;MT-TE*	**93**	8 m	NA	NA	CIV	NA	Unknown
**29**	1 y 4 m	3.4	4.0	CIV	+	*SURF1*	**94**	8 y 1 m	NA	NA	CI	-	Unknown
**30**	Perinatal	5.6	6.0	CIV	+	*SURF1*	**95**	18 y 8 m	2.4	3.1	-	-	Unknown
**31**	1 y	3.5	3.3	CIV	+	*SURF1*	**96**	1 y 1 m	NA	NA	-	-	Unknown
**32**	5 m	7.8	6.0	CIV	+	*SURF1*	**97**	3 m	3.3	NA	CIV	+	Unknown
**33**	1 y	2.6	NA	CIV	NA	*SURF1*	**98**	7 m	5.7	3.9	CI-IV	+	Unknown
**34**	2 m	NA	NA	CIV	-	*SURF1*	**99**	5 y 9 m	2.5	3.5	CI	+	Unknown
**35**	8 m	NA	NA	CIV	-	*SURF1*	**100**	1 y	12.4	1.3	-	-	Unknown
**36**	5 m	3.3	3.6	CIV	+	*SURF1*	**101**	Perinatal	8.4	6.3	CI, CIV	+	Unknown
**37**	1 m	3.0	3.2	NA	NA	*SLC19A3*	**102**	8 m	0.8	1.0	-	-	Unknown
**38**	1 m	NA	NA	NA	NA	*SLC19A3*	**103**	6 y 5 m	3.1	1.8	CI	NA	Unknown
**39**	Perinatal	2.1	NA	NA	NA	*SLC19A3*	**104**	IU	11.1	NA	NA	NA	NA
**40**	6 m	3.1	NA	-	+	*SLC19A3*	**105**	4 m	4.4	3.7	-	-	Unknown
**41**	1 m	4.4	NA	-	-	*SLC19A3*	**106**	2 y	2.9	17.0	CI, CV	+	Unknown
**42**	1 m	3.4	2.1	CIV	NA	*SLC19A3*	**107**	Perinatal	4.8	5.5	CI	-	NA
**43**	1 m	3.8	NA	NA	NA	*SLC19A3*	**108**	6 m	1.6	2.7	NA	NA	NA
**44**	2 y 7 m	3.3	1.2	-	-	*SLC19A3*	**109**	3 y	2.8	1.7	CI-IV	+	Unknown
**45**	3 m	2.0	1.7	-	-	*SLC19A3*	**110**	6 m	4.7	3.1	CI-IV	+§	Unknown
**46**	5 m	2.1	NA	-	+	*SUCLA2*	**111**	7 m	2.1	4.1	NA	NA	NA
**47**	1 m	3.0	3.3	NA	NA	*SUCLA2*	**112**	7 m	2.3	4.1	CI-IV	+	Unknown
**48**	3 m	5.9	2.9	NA	NA	*SUCLA2*	**113**	Perinatal	7.9	2.8	CI, CIV	+	Unknown
**49**	5 m	1.4	NA	NA	NA	*SUCLA2*	**114**	7 y	2.4	1.4	-	-	Unknown
**50**	2 m	NA	6.3	NA	NA	*SUCLA2*	**115**	1 m	0.8	4.4	-	-	Unknown
**51**	3 m	NA	NA	NA	NA	*SUCLA2*	**116**	3 y 8 m	5.7	5.0	-	+	Unknown
**52**	Perinatal	4.9	NA	CI, CIII, CIV	NA	*SUCLA2*	**117**	4 y	2.1	1.8	-	-	Unknown
**53**	Perinatal	5.6	3.1	CI, CIV	+	*SUCLA2*	**118**	7 y	2.3	1.3	CI	+	Unknown
**54**	IU	4.3	NA	CIV	-	*SUCLA2*	**119**	11 m	2.6	NA	-	-	Unknown
**55**	5 m	4.5	2.8	CI, CIV	-	*SUCLA2*	**120**	4 m	3.9	1.2	-	+	Unknown
**56**	7 m	5.0	2.1	CI, CIV	+	*SUCLG1*	**121**	1 y 8 m	4.2	3.1	CI	+	Unknown
**57**	3 m	5.1	4.7	CI-IV	NA	*SUCLG1*	**122**	1 y 1 m	4.3	NA	-	-	Unknown
**58**	4 y 7 m	3.3	3.7	-	-	*PDHA1*	**123**	Perinatal	NA	NA	NA	NA	Unknown
**59**	10 m	5.0	NA	-	-	*PDHA1*	**124**	10 y 5 m	3.7	NA	CI, CIII	+	Unknown
**60**	1 y 2 m	3.4	NA	NA	NA	*PDHA1*	**125**	5 m	1.3	2.8	CI	+	NA
**61**	8 m	6.2	6.2	CII	-	*PDHA1*	**126**	2 y 11 m	NA	NA	-	+	Unknown
**62**	7 m	NA	NA	NA	NA	*PDHA1*	**127**	8 y 2 m	4.2	NA	CI	-	NA
**63**	8 m	5.6	3.0	-	+	*SERAC1*	**128**	4 y 3 m	1.5	NA	CI, CIII	+	NA
**64**	Perinatal	3.8	9.1	CII, CIII	-	*SERAC1*	**129**	1 y 9 m	3.9	1.5	CI	-	Unknown
**65**	Perinatal	2.6	3.0	-	-	*SERAC1*	**130**	6 m	8.3	5.4	NA	NA	NA

*B* Blood; *CSF* Cerebrospinal fluid; *NA* Not applicable; *CI* Complex I deficiency; *CII* Complex II deficiency, *CIII* Complex III deficiency, *CIV* Complex IV deficiency; *CV* Complex V deficiency; *CI-III* Complex I to complex III deficiency; *CI-IV* Complex I to complex IV deficiency; *y* year(s); *m* month(s); *IU* intrauterine; §on electron microscopy.

Numbers in ‘bold’ represent the patient ID numbers.

Abnormal respiratory chain enzyme activity was found in 70% of examined patients, with the most prevalent being complex I deficiency. In 24 of 57 patients with abnormal histological findings in the muscle, at least one of the following was found: cytochrome c oxidase deficiency, succinate dehydrogenase deficiency, ragged red fibers and signs of abnormal mitochondrial proliferation. Genetic etiology was confirmed in 77 patients (59.2%) of which nuclear DNA mutations were much more common than mitochondrial DNA mutations (37.7% and 21.5% respectively) (Table 4).

### Acute exacerbations

The study population was followed up for a median time of 9.6 years from disease onset. In total, 56.9% of patients experienced at least one acute exacerbation requiring hospitalisation during their disease course, 43.8% during the previous year. Of these, one fourth had at least three exacerbations during the previous year. Intensive care was required in 39.2% of hospitalized patients. The main cause of acute exacerbation was infection (60.8%); other causes included respiratory complications (13.5%), stroke-like episodes (4.0%) and poor nutrition or dehydration (4.0%).

### Survival status

**Figure 4:**
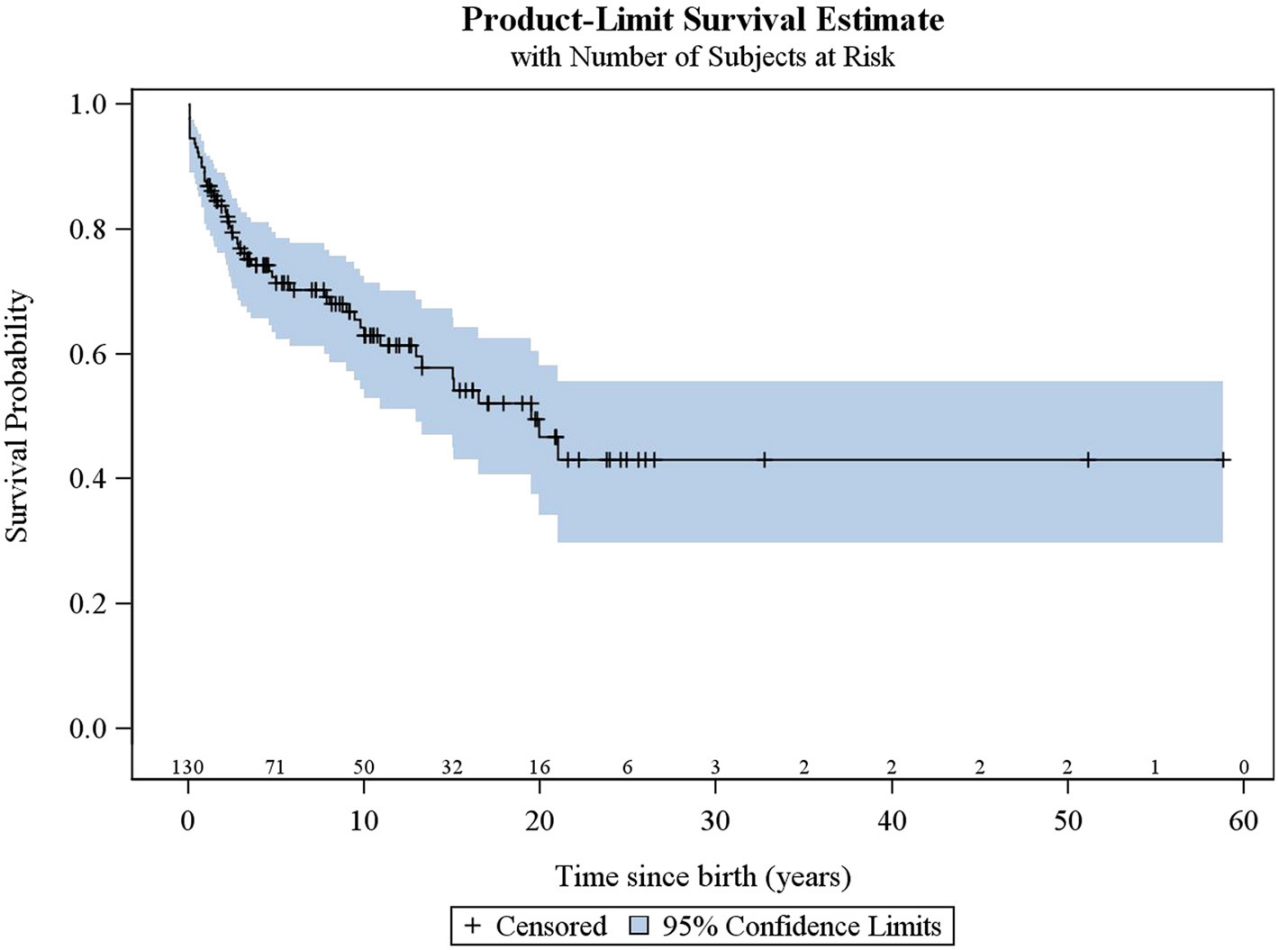
Kaplan-Meier survival curve.

53 patients were alive at the time of data analysis (40.8%), 51 were dead (39.2%) and 26 (20.0%) were lost to follow-up. Median age at death was 2.4 years (range: 1 month – 21 years). The elapsed median time from disease onset to death was 1.8 years. Main causes of death were respiratory complications (51.0%), progression of Leigh syndrome (17.6%) or infection (17.6%). The survival analysis for the study population is shown in Figure 4.

### Factors associated with acute exacerbations and/or relapses

The following factors were analysed using a univariate analysis: gender, age at onset below versus above 6 months, pathological signs at birth, complex IV deficiency, genetically verified disease, mitochondrial DNA mutations, nuclear mutations, presence of specific genetic defects (i.e. *SUCLA2; SLC19A3; SURF1; MT-ATP6*; mitochondrial NADH dehydrogenase subunits 1 to 6), muscle pathology, brainstem dysfunction on neuroimaging, basal ganglia dysfunction alone on neuroimaging and presence of any of the following clinical features - dystonia, ataxia, epileptic seizures, cardiac dysfunction, failure to thrive, hepatic dysfunction. Of these, the presence of pathological signs at birth and a history of epileptic seizures showed a significant association with higher occurrence of acute exacerbations and/or relapses. The presence of basal ganglia dysfunction alone on neuroimaging was significantly associated with lower occurrence of acute exacerbations and/or relapses.

We then tested these factors using multiple logistic regression analysis. This confirmed that the presence of pathological signs at birth and a history of epileptic seizures are the two factors significantly associated with higher occurrence of acute exacerbations and/or relapses (*p* = 0.0081 and *p* = 0.0005, respectively).

### Factors associated with survival

**Figure 5:**
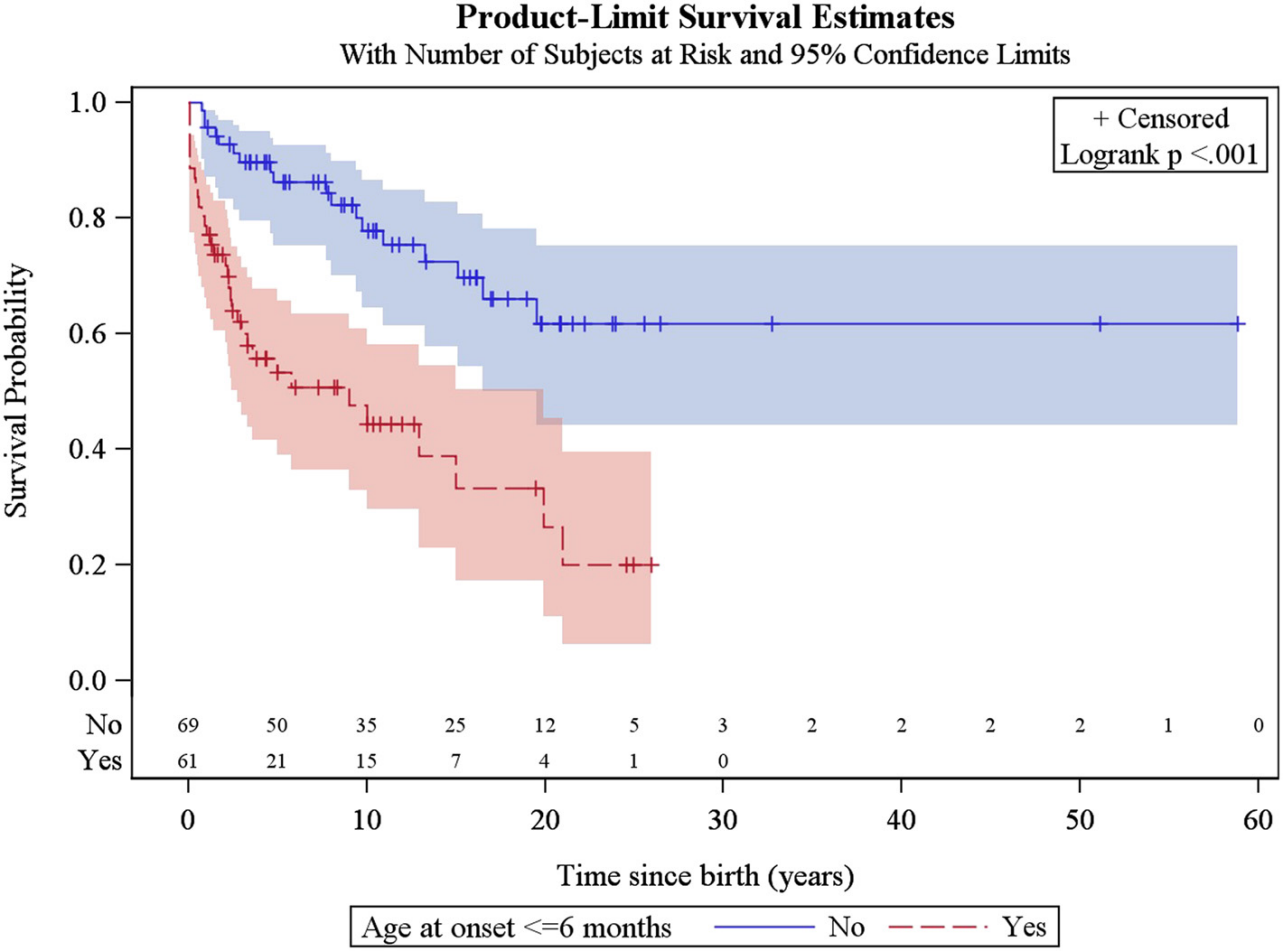
Kaplan-Meier survival curve for disease onset before versus after 6 months of age.

**Figure 6:**
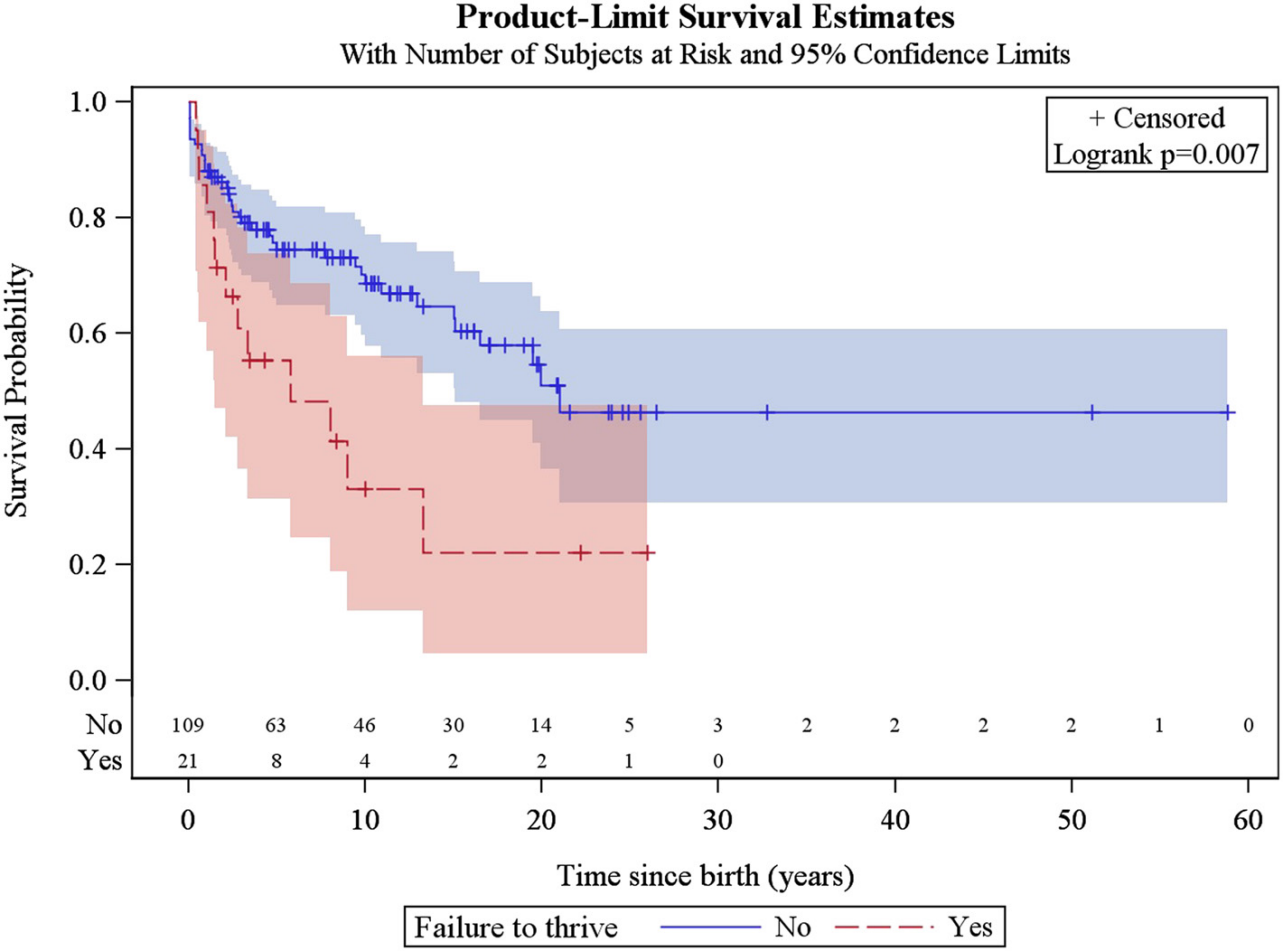
Kaplan-Meier survival curve for those patients with versus without history of failure to thrive.

**Figure 7:**
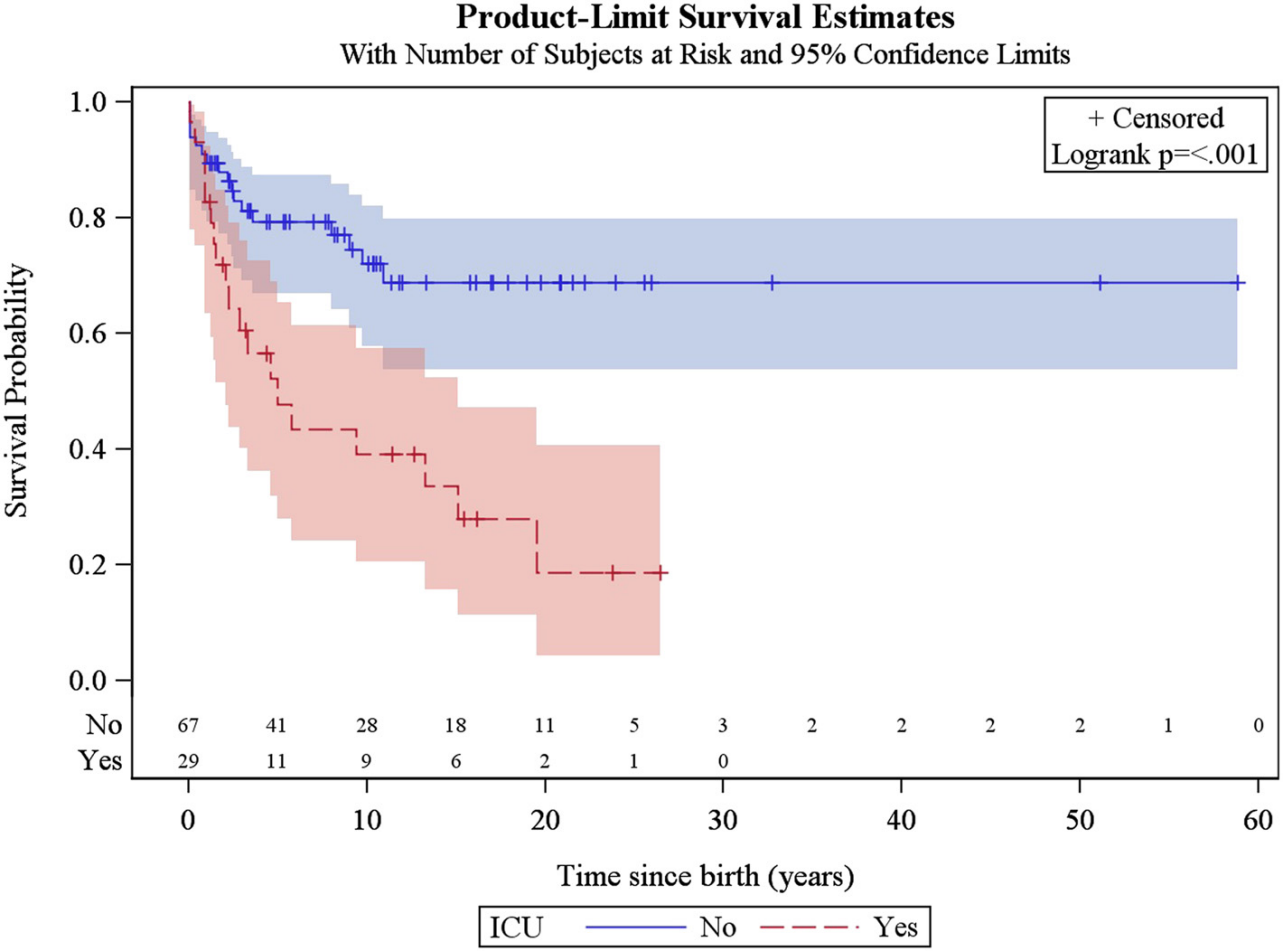
Kaplan-Meier survival curve for those patients with versus without history of treatment in intensive care unit (ICU).

**Figure 8:**
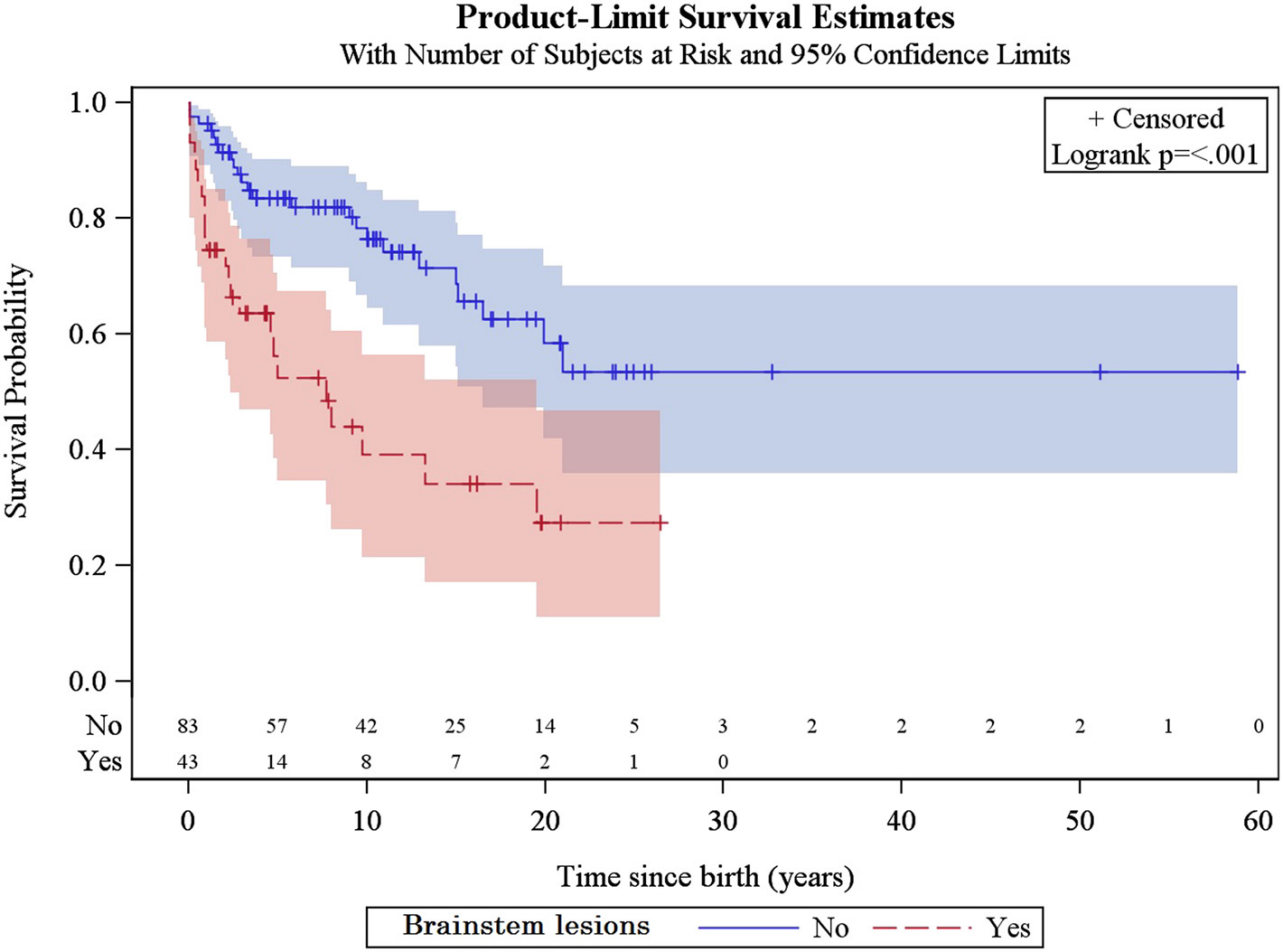
Kaplan-Meier survival curve for those patients with versus without brainstem lesions on neuroimaging.

The same factors were analysed for their association with patient survival, together with the occurrence of acute exacerbations/relapses and requirement of intensive care. The univariate analysis showed that the following were associated with poorer survival: age of onset below or equal to 6 months, history of epileptic seizures, failure to thrive, hospitalisation in intensive care unit, genetically verified disease, *SLC19A3* mutations, *mt.8993 T > G* mutation, *SURF1* mutations and brainstem lesions on neuroimaging. The multivariate analysis confirmed that age of onset before 6 months, failure to thrive, brainstem lesions on neuroimaging and requirement of intensive care are predictors of poorer survival (Figures 5, 6, 7 and 8, Additional file 3: Figure S1).

## Discussion

We present natural history data on a unique cohort of 130 patients with Leigh syndrome. Our data help define the natural history of Leigh syndrome and identify novel factors that predict outcome and long-term prognosis. Our findings are essential in order to provide optimal management and monitor the effect of future therapies.

Leigh syndrome is traditionally considered to be a disease of infancy and early childhood with disease onset before the age of 2 years [4,14-16]. In our study, we chose not to use an age-of-onset criterion, but the clinical and pathological diagnostic criteria for Leigh syndrome. This was done to better understand the age-of-onset distribution of Leigh syndrome, to identify the frequency of late-onset Leigh syndrome and differences between early and late-onset Leigh syndrome.

Prenatal expression of mitochondrial disease has been described with abnormal brain neuroimaging performed *in utero* and associated clinical features emerging at birth or during early postnatal life [17-19]. There are few reports associating abnormal intrauterine findings with postnatal development of Leigh syndrome [17,20]. The patient with Leigh syndrome reported by Kumakura et al., had intrauterine findings of oligohydramnios and intrauterine growth restriction in addition to abnormal brain neuroimaging [20]. We report two patients with abnormal intrauterine findings associated with perinatal development of Leigh syndrome. In total, pathological signs at birth were reported in 23% of our patients. However, only half of these patients were reported to have perinatal onset of Leigh syndrome. This figure is in accordance with previous reports that range from 15% [21] to 32% [15]. We found a positive correlation between the presence of pathological signs at birth and the occurrence of acute exacerbations during the disease course. The relatively high incidence of pathological signs at birth, even if not fully expressing Leigh syndrome, suggests that an ongoing energy failure linked to mitochondrial dysfunction may already have started *in utero*, and as a consequence, these patients might be more susceptible to external stress factors, such as infection, poor nutrition and dehydration, leading to acute exacerbations later in the disease course. The factors that regulate the expression time –from *in utero* to adulthood- of this mitochondrial energy failure remain to be understood.

Late-onset Leigh syndrome, i.e. presentation in late childhood, adolescence or adulthood, is considered rare. In our study, however, 19.2% of the patients had onset after 2 years of age, with the latest onset (10%) after 4.9 years of age. Late-onset Leigh syndrome has been associated with predominant extrapyramidal features, slow progression and acute deterioration, but atypical presentations have also been described [9]. In their review of late-onset Leigh syndrome, McKelvie et al.*,* presented 13 patients with onset of Leigh syndrome between 17 and 74 years [22]. These patients had highly variable clinical features, the most prominent being ataxia, spasticity, dysarthria and abnormal ocular findings. Prognosis was observed to be poor with 10 of 13 patients dying within 2 weeks to 6 years from disease onset [22]. In contrast, of the 13 patients with the latest onset presented in our study (10%), all but two patients were alive. There was a high prevalence of dystonia and ataxia in this subgroup of patients, suggesting that such involvement might be age dependent. The heterogeneity of the phenotypic spectrum and of the disease course of Leigh syndrome makes it difficult to define an age cut-off in order to identify late-onset Leigh syndrome. However, based on the age-of-onset distribution of our patients, we propose the age of 2 years as a cut-off to distinguish between early versus late-onset Leigh syndrome.

While Leigh syndrome is not primarily a disease affecting the cerebral cortex, 40% of our patients developed epileptic seizures. The reported prevalence of seizures in Leigh syndrome ranges from 40% to 79% [4,14,15,23]. In addition, we found a significantly higher occurrence of acute exacerbations and/or relapses in patients with a history of seizures. However, seizures were the cause of exacerbations and/or relapses in only three patients. The relapse occurrence was attributed to infections in the vast majority of our patients with history of seizures. Our findings suggest that patients with Leigh syndrome and a history of seizures are at risk of acute exacerbations and have increased need for hospitalisation when infected.

The survival rate in Leigh syndrome is generally considered to be poor. Rahman et al.*,* reported a survival of 20% by the age of 20 years, with death typically occurring by age 2 to 3 years [4]. In the study by Lee et al., eight of 14 patients (57%) had died by the age of 16 years, seven of whom by the age of 18 months [14]. Poor prognosis was reported by Naess et al.; 68% of patients died by the age of 15 years, 40% of patients at preschool age [15]. Our study population was found to have a better survival rate, with 41% of patients being alive at a median follow-up of 11.4 years from birth (range: 0.1 – 58 years). However, in the 39% of patients that had died by the age of 21 years, an early mortality was found at a median age of 2.4 years.

The natural history of Leigh syndrome has been described for the subgroups of *SURF1* deficiency, cytochrome c oxidase deficiency due to *LRPPRC* mutations and complex I deficiency [24-26]. In particular, *SURF1*-deficient Leigh syndrome was found to have a more favorable survival outcome compared to Leigh syndrome associated with complex I-deficiency or *LRPPRC* mutations [24].

Predictors of survival in Leigh syndrome have not previously been assessed, probably due to the small numbers of patients per study. We found disease onset before 6 months of age, failure to thrive, brainstem lesions on neuroimaging and intensive care treatment to be associated with poorer survival. Poor survival in patients with onset before 6 months of age has been previously reported, not only in Leigh syndrome [15] but in mitochondrial disorders in general [1]. The effect of brainstem dysfunction on patient outcome has been studied in 130 patients with complex I deficiency, half of them with the diagnosis of Leigh syndrome, and no correlation was found [26]. In our study, respiratory complications were found to be the leading cause of death, with half of our patients dying of this complication. In view of the significant correlation between brainstem lesions on neuroimaging and poor survival, we believe that brainstem involvement may also be responsible for the respiratory complications seen in patients with Leigh syndrome. Patients treated in intensive care unit appear to have a higher risk of death. As respiratory complications and disease progression accounted for the majority of deaths, we believe that it is the disease severity with signs of progressive brainstem involvement related to the respiratory insufficiency, of the patients admitted to intensive care unit that mainly leads to increased mortality.

Cardiomyopathy has been associated with poor survival in mitochondrial diseases [27]. In a study of 101 patients with all types of mitochondrial disorders, cardiomyopathy was found in 17% of patients and was associated with increased mortality [27]. Cardiac dysfunction was found in 18% of our patients. This is almost as high as that reported by Lee et al. [14], but higher than in other studies [4,15,23]. Among the 23 patients with cardiac dysfunction included in our study, 11 patients had died by the time of data capturing, 9 patients were alive and 3 patients were lost to follow-up. Cardiac dysfunction was not associated with poor survival or increased risk of disease relapse in our patients.

Rahman et al.*,* included the presence of increased lactate in either blood or CSF among the diagnostic criteria for Leigh syndrome [4], a criterion that has been questioned [15]. In our study, 25% of the patients with available lactate values either in blood or CSF had normal levels throughout the disease course. Of these, 10 patients had genetically verified disease. We conclude, therefore, that the clinical and pathological inclusion criteria used in our study, which do not include any laboratory parameters, are more appropriate as diagnostic criteria for Leigh syndrome. Increased lactate in CSF was significantly correlated to a more severe disease course in our patients, characterised by early onset before 6 months of age, acute exacerbations and/or relapses, as well as brainstem involvement. We believe, therefore that the presence of abnormal lactate in CSF may be used as a prognostic factor of disease severity in Leigh syndrome.

A limitation of the present paper is that all participating centers are located in Northern and Western Europe, and as a result, the majority of patients have a specific genetic background which may determine differences in other regions of the world.

Leigh syndrome should be differentiated from other diseases associated with degeneration of the basal ganglia in the paediatric population, such as carbon monoxide intoxication, infantile bilateral striatal necrosis (IBSN), Wilson’s disease, juvenile Huntington’s chorea, neurogeneration with brain iron accumulation, familial degeneration of the striatum and GAMT (guanidinoacetate methyltransferase) deficiency. These diseases can generally be ruled out on the basis of laboratory tests, history of exposure to toxins and family history. IBSN includes a heterogeneous group of disorders, characterised by bilateral symmetrical degeneration predominantly of the caudate nuclei and the putamen. Etiologically, IBSN has been attributed to infectious and autoimmune processes, while a mitochondrial dysfunction has also been suggested in familial cases [28]. Non-familial IBSN differs from Leigh syndrome in the absence of a chronic progressive course [28,29]. In view of the fact that familial IBSN shows a phenotypic spectrum that overlaps with that of Leigh syndrome with lesions restricted to the striatum, we suggest that primary or secondary defects of the mitochondrial OXPHOS should be considered.

At least 3.1 years elapsed from disease onset to diagnostic testing for suspected mitochondrial disease in 25% of our patients with Leigh syndrome. A delay in diagnosis is common in mitochondrial diseases. In a case series by Lee et al.*,* on 21 patients with *SURF1*-associated Leigh syndrome, the median age at the time of molecular diagnosis was 4.1 years with a range from 1.2 to 22.0 years [30]. A better understanding of the phenotypic spectrum and natural history of Leigh syndrome can lead to earlier diagnosis and improved counseling to the family, including prenatal counseling. The identification of genotype-phenotype and genotype-neuroimaging correlations is essential and it is the next step of our research work.

## Conclusions

Leigh syndrome is a genetically heterogeneous disorder characterised by a vast spectrum of phenotypes and a variable disease course. We present longitudinal natural history data from a unique cohort of 130 patients with Leigh syndrome. We discuss the diagnostic criteria of Leigh syndrome and propose novel predictors of disease severity and long-term prognosis. Our findings are essential in order to provide optimal management and monitor the effect of future therapies.

### Availability of supporting data

The data sets supporting the results of this article are included within the article and its additional files.

## Abbreviations

CRF: Case report form; CT: Computed tomography; IBSN: Infantile bilateral striatal necrosis; MCRN: Mitochondrial Clinical and Research Network; MRI: Magnetic resonance imaging; OXPHOS: Oxidative phosphorylation; PDHc: Pyruvate dehydrogenase complex.

## Competing interests

The authors declare that they have no competing interests.

## Authors’ contributions

KS coordinated the study and drafted the manuscript. ND and MT supervised the study. KS, ND and MT analysed the data. All authors participated in the concept and design of the study, contributed to the acquisition and interpretation of data and helped to revise the manuscript. All authors read and approved the final manuscript.

## Supplementary Material

Additional file 1: Table S1 Case Report Form. Click here for file

Additional file 2: Table S2 Abnormal motor findings at disease onset versus later. Click here for file

Additional file 3: Figure S1 Predictors of survival. Click here for file
