# Supplementary material for: A multicenter study on Leigh syndrome: disease course and predictors of survival
Source: Orphanet J Rare Dis. 2014 Apr 15;9:52. doi: 10.1186/1750-1172-9-52 (PMC4021638; doi:10.1186/1750-1172-9-52)
Supplement: Additional file 1: Table S1 — Case Report Form. [file 1750-1172-9-52-S1.pdf]

CASE REPORT FORM (CRF)
A Retrospective, Multinational Study of Patients with Leigh Syndrome

Site ID: __ __	Patient ID: __ __ __ __	Date of registration: __/__/_______

INCLUSION/EXCLUSION CRITERIA

INCLUSION CRITERIA		
1. Clinical features compatible with Leigh syndrome, i.e. psychomotor regression, brainstem dysfunction, ataxia, dystonia, optic atrophy	☐ **Yes**	☐ No
2. MRI or CT or neuropathological findings of Leigh syndrome, i.e. bilateral symmetrical lesions in the basal ganglia, and/or thalamus, and/or brainstem§.	☐ **Yes**	☐ No
All inclusion criteria must be answered **Yes**, to be included in the study.		
**EXCLUSION CRITERIA**		
1. Known syndromic mitochondrial phenotype other than Leigh syndrome.	☐ Yes	☐ **No**
All exclusion criteria must be answered **No**, to be included in the study. If any of the exclusion criteria is answered Yes, the patient must be excluded from the study and no data must be collected in the following fields of the present CRF.		

DEMOGRAPHICS

- **Date of birth** (mm/yyyy): __/_______
- **Gender**: ☐ male ☐ female
- **Race**: ☐ caucasian ☐ black ☐ asian ☐ other__________ ☐ Unknown

FAMILY HISTORY

- **Parental consanguinity**: ☐ No ☐ Yes ☐ Unknown
  If yes, specify degree of relatedness: ________________
- **Presence of Leigh (or Leigh-like) syndrome in a relative**: ☐ No ☐ Yes ☐ Unknown
  If yes, specify a) gender: ☐ Male ☐ Female ☐ Unknown
  b) origin of relatedness: ☐ Maternal ☐ Paternal ☐ Unknown
  c) degree of relatedness: ☐ 1st-degree ☐ 2nd-degree ☐ 3rd-degree ☐ >3rd-degree ☐ Unknown

- **Presence of other mitochondrial disorder in a relative**: ☐ No ☐ Yes ☐ Unknown
  If yes, specify a) Disorder_______________________,
  b) Origin of relatedness: ☐ Maternal ☐ Paternal ☐ Unknown
  c) Degree of relatedness: ☐ 1st-degree ☐ 2nd-degree ☐ 3rd-degree ☐ >3rd-degree ☐ Unknown

- **Presence of other neurological disorder in a relative**: ☐ No ☐ Yes ☐ Unknown
  If yes, specify a) Disorder_______________________,
  b) Origin of relatedness: ☐ Maternal ☐ Paternal ☐ Unknown
  c) Degree of relatedness: ☐ 1st-degree ☐ 2nd-degree ☐ 3rd-degree ☐ >3rd-degree ☐ Unknown
- **Comments** ____

MEDICAL HISTORY

Perinatal History

- **Gestational age**: ☐ <35 weeks ☐ 35-37 weeks ☐ 38-41 weeks ☐ >41 weeks ☐ Unknown
- **Pregnancy**: ☐ uneventful ☐ complicated by ______________________ ☐ Unknown
- **Birth weight**§: ☐ AGA ☐ SGA ☐ LGA ☐ Unknown
- **Did the fetus present intra-uterine growth restriction (IUGR)**§: ☐ Yes ☐ No ☐ Unknown
- **Head circumference at birth**§: ☐ normal ☐ microcephaly ☐ macrocephaly ☐ Unknown
- **APGAR score at 1-5-10 minutes**: ☐ - ☐ - ☐ ☐ Unknown
- **Respiratory difficulties at birth necessitating interventions**: ☐ No ☐ Yes ☐ Unknown
  If yes, specify: ☐ oxygen by mask ☐ nasal CPAP ☐ intubation/ventilation
- **Any pathological signs at birth**: ☐ No ☐ Yes ☐ Unknown
  If yes, specify: ☐ hypotonia/floppiness ☐ hypertonia ☐ respiratory complications ☐ seizures
  ☐ dysmorphic features ☐ cardiac complications ☐ congenital lactic acidosis
  ☐ other: ___________
- **Prenatal onset of symptoms**: ☐ Yes ☐ No ☐ Unknown
  If no, date at onset of symptoms (mm/yyyy): __/_____
- **Date of diagnostic testing for suspected mitochondrial disease** (mm/yyyy): __/_____

§ *see Attachment I: Instructions for Investigator*

- **Specify type of diagnostic testing** (more than one answers may be selected):
☐ muscle biopsy ☐ liver biopsy ☐ fibroblasts ☐ genetic investigation ☐ other, specify________

Clinical Features				
Fill in any of the following clinical features presented either at onset or during the disease course and specify time at presentation (i.e. at onset or later).				
**Motor dysfunction** ☐No ☐ Yes		*If yes, please specify all that are applicable:*		
☐Hypotonia		☐At onset	☐later	☐Unknown
☐Hypertonia		☐At onset	☐later	☐Unknown
☐Dystonia		☐At onset	☐later	☐Unknown
☐Ataxia				
	☐*Cerebellar*	☐At onset	☐later	☐Unknown
	☐*Sensory*	☐At onset	☐later	☐Unknown
	☐*Unknown*			
☐Spasticity		☐At onset	☐later	☐Unknown
☐Paresis/palsy				
	☐*Hemiparesis/plegia*	☐At onset	☐later	☐Unknown
	☐*Paraparesis/plegia*	☐At onset	☐later	☐Unknown
	☐*Quadriparesis/plegia*	☐At onset	☐later	☐Unknown
	☐*Monoparesis/plegia*	☐At onset	☐later	☐Unknown
☐Myoclonus		☐At onset	☐later	☐Unknown
☐Hypokinesia/ Bradykinesia		☐At onset	☐later	☐Unknown
☐Chorea/Athetosis		☐At onset	☐later	☐Unknown
☐Other dyskinetic disorder				
Specify:	____________________	☐At onset	☐later	☐Unknown
☐Muscle weakness		☐At onset	☐later	☐Unknown
☐Cavus feet		☐At onset	☐later	☐Unknown
☐Babinski sign		☐At onset	☐later	☐Unknown
☐Tendon reflexes				
Specify:	____________________	☐At onset	☐later	☐Unknown
**Ophthalmological dysfunction** ☐No ☐ Yes		*If yes, please specify all that are applicable:*		
☐Ptosis		☐At onset	☐later	☐Unknown
☐Reduced eye motility		☐At onset	☐later	☐Unknown
☐Strabismus		☐At onset	☐later	☐Unknown
☐Nystagmus		☐At onset	☐later	☐Unknown
☐Optic atrophy		☐At onset	☐later	☐Unknown
☐Retinopathy		☐At onset	☐later	☐Unknown
☐Visual impairment		☐At onset	☐later	☐Unknown
☐Other				
Specify:	___________________	☐At onset	☐later	☐Unknown

**Other dysfunction** ☐No ☐ Yes		*If yes, please specify all that are applicable:*		
☐Seizures				
	☐*Partial seizures*	☐At onset	☐later	☐Unknown
	☐*Generalized seizures*	☐At onset	☐later	☐Unknown
	☐*Neonatal seizures*	☐At onset	☐later	☐Unknown
	☐*Myoclonic seizures*	☐At onset	☐later	☐Unknown
	☐*Epilepsy syndrome*____________	☐At onset	☐later	☐Unknown
	☐*Other* ____________	☐At onset	☐later	☐Unknown
Are seizures therapy-resistant?		☐ Yes	☐ No	
Has the patient received valproate treatment?		☐ Yes	☐ No	
☐Cardiac dysfunction				
	☐*Conduction defects*	☐At onset	☐later	☐Unknown
	☐*Dilated cardiomyopathy*	☐At onset	☐later	☐Unknown
	☐*Hypertrophic cardiomyopathy*	☐At onset	☐later	☐Unknown
	☐*Other* ____________	☐At onset	☐later	☐Unknown
☐Hepatic dysfunction				
	Specify: ____________________	☐At onset	☐later	☐Unknown
☐Renal dysfunction		☐At onset	☐later	☐Unknown
	Specify: ____________________	☐At onset	☐later	☐Unknown
☐Failure to thrive§		☐At onset	☐later	☐Unknown
☐Feeding difficulties				
	☐requiring gastrostomi	☐At onset	☐later	☐Unknown
	☐requiring feeding tube	☐At onset	☐later	☐Unknown
☐Sucking dysfunction		☐At onset	☐later	☐Unknown
☐Mental retardation§				
	☐*Mild*	☐At onset	☐later	☐Unknown
	☐*Moderate*	☐At onset	☐later	☐Unknown
	☐*Severe*	☐At onset	☐later	☐Unknown
	☐*Profound*	☐At onset	☐later	☐Unknown
☐Microcephaly		☐At onset	☐later	☐Unknown
	Progressive microcephaly§ ☐yes ☐no			
☐Hearing dysfunction				
	☐Conductive	☐At onset	☐later	☐Unknown
	☐Sensorineural	☐At onset	☐later	☐Unknown
☐Peripheral neuropathy				
	☐Demyelinating	☐At onset	☐later	☐Unknown
	☐Axonal	☐At onset	☐later	☐Unknown
☐Respiratory disturbance				
	☐*Apnea*	☐At onset	☐later	☐Unknown
	☐*Central hypoventilation*	☐At onset	☐later	☐Unknown
	☐*Hyperventilation/Abnormal breathing pattern*	☐At onset	☐later	☐Unknown
	☐*Respiratory complications (non-CNS induced)*	☐At onset	☐later	☐Unknown
☐Hematological dysfunction				
	Specify: ____________________	☐At onset	☐later	☐Unknown
☐Other dysfunction				
	Specify: ____________________	☐At onset	☐later	☐Unknown

§ *see Attachment I: Instructions for Investigator*

DISEASE COURSE

-Has the patient experienced acute exacerbation(s)/relapse(s) during the disease course§: ☐Yes ☐No ☐Unknown

If yes, specify **a) the frequency of acute exacerbations/relapses during the past year:**

☐ ≤ 2 times ☐ 3-5 times ☐ 6-10 times ☐ >10 times ☐ Unknown

b) the main cause of acute exacerbations/relapses during the past year:

☐ infection(s) ☐ other, specify:______ ☐ Unknown

c) did any of the acute exacerbations/relapses require ICU: ☐ Yes ☐ No ☐ Unknown

SURVIVAL STATUS

- **Current status**§: ☐ Alive ☐ Deceased ☐ Unknown/Lost-to-follow-up

If lost-to-follow-up, please specify last date of follow-up (mm/yyyy): __/____

If deceased, please specify§: **a) Date of death**: __/____

b) Cause of death: ________________________

c) Has postmortem investigation been performed: ☐ Yes ☐ No ☐ Unknown

GROWTH§

- Date of latest examination (mm/yyyy): __/____	☐ Unknown
- Weight for age: ___ SD	☐ Unknown
- Weight for height: ___ SD	☐ Unknown
- Height for age: ___ SD	☐ Unknown
- Head circumference for age: ___ SD	☐ Unknown

LABORATORY FINDINGS

Respiratory chain enzyme activity
(more than one fields may be selected)

☐Compex I deficiency ☐Compex II deficiency ☐Compex III deficiency ☐Compex IV deficiency ☐Compex V deficiency

**Absolute values in blood§ (units)**			
Lactate	______________	mmol/l	☐ Unknown
Pyruvate	______________	mmol/l	☐ Unknown
Albumin	______________	g/l	☐ Unknown
**Absolute values in CSF§ (units)**			
Lactate	______________	mmol/l	☐ Unknown
Pyruvate	______________	mmol/l	☐ Unknown
Albumin	______________	mg/l	☐ Unknown
**Lactate: pyruvate ratio in blood§ (units)**			
Lactate/Pyruvate	___________ mmol/l / ________	mmol/l	☐ Unknown
**Lactate: pyruvate ratio in CSF § (units)**			
Lactate/Pyruvate	___________ mmol/l / ________	mmol/l	☐ Unknown
**CSF/serum albumin ratio§** _____________			☐ Unknown

**Pathological aminoacids in plasma** ☐Yes ☐No ☐Unknown,				If yes Specify:
1.______________	Type:______________	Absolute value: ______	Units: ______	Reference range: ____ - _____
2.______________	Type:______________	Absolute value: ______	Units: ______	Reference range: ____ - _____
3.______________	Type:______________	Absolute value: ______	Units: ______	Reference range: ____ - _____
**Pathological aminoacids in urin** ☐Yes ☐No ☐Unknown,				If yes Specify:
1.______________	Type:______________	Absolute value: ______	Units: ______	Reference range: ____ - _____
2.______________	Type:______________	Absolute value: ______	Units: ______	Reference range: ____ - _____
3.______________	Type:______________	Absolute value: ______	Units: ______	Reference range: ____ - _____
**Pathological aminoacids in CSF** ☐Yes ☐No ☐Unknown,				If yes Specify:
1.______________	Type:______________	Absolute value: ______	Units: ______	Reference range: ____ - _____
2.______________	Type:______________	Absolute value: ______	Units: ______	Reference range: ____ - _____
3.______________	Type:______________	Absolute value: ______	Units: ______	Reference range: ____ - _____
**Pathological organic acids in urin** ☐Yes ☐No ☐Unknown,				If yes Specify:
1.______________	Type:______________	Absolute value: ______	Units: ______	Reference range: ____ - _____
2.______________	Type:______________	Absolute value: ______	Units: ______	Reference range: ____ - _____
3.______________	Type:______________	Absolute value: ______	Units: ______	Reference range: ____ - _____

§ see Attachment I: Instructions for Investigator

LABORATORY FINDINGS (continued)				
**Pathological total or free carnitine**	☐Yes	☐No	☐Unknown,	If yes Specify:
Type:________________ Tissue:______________	Absolute value: ______	Units: ______	Reference range: ____ - _____	
**Pathological acylcarnitine**	☐Yes	☐No	☐Unknown,	If yes Specify:
Type:________________ Tissue:______________	Absolute value: ______	Units: ______	Reference range: ____ - _____	
**Pathological Q10**	☐Yes	☐No	☐Unknown,	If yes Specify:
Type:________________ Tissue:______________	Absolute value: ______	Units: ______	Reference range: ____ - _____	
**Pathological a-fetoprotein**	☐Yes	☐No	☐Unknown,	If yes Specify:
Type:________________ Tissue:______________	Absolute value: ______	Units: ______	Reference range: ____ - _____	
**Pyruvate dehydrogenase complex deficiency**	☐Yes	☐No	☐Unknown	
**Pyruvate carboxylase deficiency**	☐Yes	☐No	☐Unknown	
**Krebs cycle enzyme deficiency**	☐Yes Specify:______________________	☐No	☐Unknown	
**Other enzyme deficiency**	☐Yes Specify:______________________	☐No	☐Unknown	

Blue native gel performed: ☐ Yes ☐ No ☐ Unknown
If yes, specify tissue and findings: __________________

Comments:

MUSCLE PATHOLOGY

☐ Normal ☐ Abnormal
If abnormal, specify: ☐ COX deficiency ☐ SDH deficiency ☐ Ragged Red Fibers
☐ Mitochondrial proliferation ☐ Other, specify: _________

- **Electron microscopy performed**: ☐ Yes ☐ No ☐ Unknown
If yes, specify findings: __________________

LIVER PATHOLOGY

☐ Normal ☐ Abnormal ☐ Unknown
If abnormal, specify: ☐ Inflammation ☐ Steatosis ☐ Fibrosis ☐ Other, specify: ___________

GENETIC INVESTIGATIONS

- **Has the patient undergone genetic investigation(s)?** ☐Yes ☐No ☐Unknown, If yes Specify:
a) the type of genetic investigation(s) performed:____
b) the genetic findings herebelow:

- **Does the patient harbor a pathogenic mutation?** ☐ Yes ☐ No ☐ Unknown
If yes specify below, including the locus, grade of heteroplasmy and tissue where applicable:

☐MTND1	☐ Not Performed	____________________________
☐MTND3	☐ Not Performed	____________________________
☐MTND4	☐ Not Performed	____________________________
☐MTND5	☐ Not Performed	____________________________
☐MTND6	☐ Not Performed	____________________________
☐MTATP6	☐ Not Performed	____________________________
☐MTTL1	☐ Not Performed	____________________________
☐MTTK	☐ Not Performed	____________________________
☐MTCO3	☐ Not Performed	____________________________
☐Other mitochondrial gene: _________		____________________________
☐SURF1	☐ Not Performed	____________________________
☐NDUFV1	☐ Not Performed	____________________________
☐NDUFS	☐ Not Performed	____________________________
☐SUCLA2	☐ Not Performed	____________________________
☐SUCLG1	☐ Not Performed	____________________________
☐PDHA1	☐ Not Performed	____________________________
☐Other nuclear gene: _________		____________________________

- **Does the patient harbor a mtDNA depletion?** ☐ Yes ☐ No ☐ Unknown
If yes, specify: a) the method used:_______________________________________
b) the percentage of abnormal mtDNA:_________________________%
c) the corresponding tissue:____________________________________

MRI FINDINGS

<u>1st MRI</u>*:* Has MRI been done: ☐ Done ☐ Unknown/Not done

If Done, a) Date of 1st MRI§: __/_____

b) ☐ **Normal** ☐ **Abnormal**

If abnormal, specify the anatomical region(s) and type(s) of abnormalities:

Region	Signal abnormality			Atrophy	Comments
☐ Putamen	☐ Increased T1<br>☐ Decreased T1	☐ Increased T2<br>☐ Decreased T2	☐ Bilateral<br>☐ Unilateral	☐ Bilateral<br>☐ Unilateral<br>☐ No Atrophy	
☐ Caudate nucleus	☐ Increased T1<br>☐ Decreased T1	☐ Increased T2<br>☐ Decreased T2	☐ Bilateral<br>☐ Unilateral	☐ Bilateral<br>☐ Unilateral<br>☐ No Atrophy	
☐ Globus pallidus	☐ Increased T1<br>☐ Decreased T1	☐ Increased T2<br>☐ Decreased T2	☐ Bilateral<br>☐ Unilateral	☐ Bilateral<br>☐ Unilateral<br>☐ No Atrophy	
☐ Thalamus	☐ Increased T1<br>☐ Decreased T1	☐ Increased T2<br>☐ Decreased T2	☐ Bilateral<br>☐ Unilateral	☐ Bilateral<br>☐ Unilateral<br>☐ No Atrophy	
☐ N subthalamicus	☐ Increased T1<br>☐ Decreased T1	☐ Increased T2<br>☐ Decreased T2	☐ Bilateral<br>☐ Unilateral	☐ Bilateral<br>☐ Unilateral<br>☐ No Atrophy	
☐ Midbrain	☐ Increased T1<br>☐ Decreased T1	☐ Increased T2<br>☐ Decreased T2	☐ Bilateral<br>☐ Unilateral	☐ Bilateral<br>☐ Unilateral<br>☐ No Atrophy	
☐ Pons	☐ Increased T1<br>☐ Decreased T1	☐ Increased T2<br>☐ Decreased T2	☐ Bilateral<br>☐ Unilateral	☐ Bilateral<br>☐ Unilateral<br>☐ No Atrophy	
☐ Medulla	☐ Increased T1<br>☐ Decreased T1	☐ Increased T2<br>☐ Decreased T2	☐ Bilateral<br>☐ Unilateral	☐ Bilateral<br>☐ Unilateral<br>☐ No Atrophy	
☐ Cerebellum					
☐ *grey matter*	☐ Increased T1<br>☐ Decreased T1	☐ Increased T2<br>☐ Decreased T2	☐ Bilateral<br>☐ Unilateral	☐ Bilateral<br>☐ Unilateral<br>☐ No Atrophy	
☐ *white matter*	☐ Increased T1<br>☐ Decreased T1	☐ Increased T2<br>☐ Decreased T2	☐ Bilateral<br>☐ Unilateral	☐ Bilateral<br>☐ Unilateral<br>☐ No Atrophy	
☐ *vermis*	☐ Increased T1<br>☐ Decreased T1	☐ Increased T2<br>☐ Decreased T2	☐ Bilateral<br>☐ Unilateral	☐ Bilateral<br>☐ Unilateral<br>☐ No Atrophy	
☐ *peduncles*	☐ Increased T1<br>☐ Decreased T1	☐ Increased T2<br>☐ Decreased T2	☐ Bilateral<br>☐ Unilateral	☐ Bilateral<br>☐ Unilateral<br>☐ No Atrophy	
☐ *dentate nucleus*	☐ Increased T1<br>☐ Decreased T1	☐ Increased T2<br>☐ Decreased T2	☐ Bilateral<br>☐ Unilateral	☐ Bilateral<br>☐ Unilateral<br>☐ No Atrophy	
☐ Cerebral cortex	☐ Increased T1<br>☐ Decreased T1	☐ Increased T2<br>☐ Decreased T2	☐ Bilateral<br>☐ Unilateral	☐ Bilateral<br>☐ Unilateral<br>☐ No Atrophy	
☐ Supratentorial white matter	☐ Increased T1<br>☐ Decreased T1	☐ Increased T2<br>☐ Decreased T2	☐ Bilateral<br>☐ Unilateral	☐ Bilateral<br>☐ Unilateral<br>☐ No Atrophy	
☐ Corpus callosum	☐ Increased T1<br>☐ Decreased T1	☐ Increased T2<br>☐ Decreased T2	☐ Bilateral<br>☐ Unilateral	☐ Atrophy/hypoplasia<br>☐ Agenesis<br>☐ No Atrophy	
☐ Additional/other MRI findings: ______________________________					

- **Diffusion-weighted imaging (DWI):** ☐ Normal ☐ Abnormal ☐ Unknown/Not done

If Normal, specify timepoint after acute episode (in days): ____ days

If abnormal, specify: timepoint after acute episode (in days): ____ days

a) type of abnormalities: ______________ b) region of abnormalities: _________________

- **Apparent diffusion coefficient (ADC):** ☐ Normal ☐ Abnormal ☐ Unknown/Not done

If abnormal, specify a) type of abnormalities: __________________

b) region of abnormalities: ________________

- **Magnetic Resonance Spectroscopy (MRS):** ☐ Normal ☐ Abnormal ☐ Unknown/Not done

If abnormal, specify type and region of abnormalities

☐ Elevated lactate Region: ______________ ☐ Decreased NAA Region: ______________

☐ Elevated choline Region: ______________ ☐ Other, specify ______________ Region: ______________

☐ Elevated succinate Region: ______________

§ *see Attachment I: Instructions for Investigator*

2nd MRI: Has 2nd MRI been done: ☐ Done ☐ Unknown/Not done
If Done, specify Date of 2nd MRI§: __/_____
If 2nd MRI available, specify the progress of MRI findings in relation to the previous MRI:
☐ Progression, specify: ___________________________
☐ Regression, specify: ____________________________
☐ No change

3rd MRI: Has 3rd MRI been done: ☐ Done ☐ Unknown/Not done
If Done, specify Date of 3rd MRI§: __/_____
If 3rd MRI available, specify the progress of MRI findings in relation to the previous MRI:
☐ Progression, specify: ___________________________
☐ Regression, specify: ____________________________
☐ No change

- Do you wish to provide illustrative MRI image: ☐ Yes ☐ No ☐ NA

CT FINDINGS

Has CT been done: ☐ Done ☐ Unknown/Not done
If Done, specify Date of 1st CT§: __/_____
If 1st CT available, specify CT findings: ☐ Normal ☐ Abnormal
If abnormal, specify the anatomical region(s) and type(s) of abnormalities:
☐ Calcification(s) Specify region: _____
☐ High attenuation Specify region: _____
☐ Low attenuation Specify region: _____
☐ Other, specify a) type___________________
b) region: _________________

TREATMENT

Does the patient receive or has the patient received any of the following treatments? ☐No ☐Yes
If Yes, specify type and status (on-going or discontinued).

☐**Antiepileptic treatment**
☐Single antiepileptic drug ☐Combination of antiepileptic drugs ☐Valproate either alone or in combination
☐On-going *specify effect of this treatment*: ☐No apparent ☐Positive effect ☐Unknown
☐Discontinued *specify reason(s) for discontinuation*: ☐Lack of efficacy ☐Non-compliance
☐Lack of safety ☐ Other: _________
☐Unknown

☐**Ketogenic diet**
☐On-going *specify effect of this treatment*: ☐No apparent ☐Positive effect ☐Unknown
☐Discontinued *specify reason(s) for discontinuation*: ☐Lack of efficacy ☐Non-compliance
☐Lack of safety ☐ Other: _________
☐Unknown

☐**Coenzyme Q1**
☐On-going *specify effect of this treatment*: ☐No apparent ☐Positive effect ☐Unknown
☐Discontinued *specify reason(s) for discontinuation*: ☐Lack of efficacy ☐Non-compliance
☐Lack of safety ☐ Other: _________
☐Unknown

☐**Thiamine/Vitamine B1**
☐On-going *specify effect of this treatment*: ☐No apparent ☐Positive effect ☐Unknown
☐Discontinued *specify reason(s) for discontinuation*: ☐Lack of efficacy ☐Non-compliance
☐Lack of safety ☐ Other: _________
☐Unknown

☐**Riboflavin**
☐On-going *specify effect of this treatment*: ☐No apparent ☐Positive effect ☐Unknown
☐Discontinued *specify reason(s) for discontinuation*: ☐Lack of efficacy ☐Non-compliance
☐Lack of safety ☐ Other: _________
☐Unknown

☐**Idebenone**
☐On-going *specify effect of this treatment*: ☐No apparent ☐Positive effect ☐Unknown
☐Discontinued *specify reason(s) for discontinuation*: ☐Lack of efficacy ☐Non-compliance
☐Lack of safety ☐ Other: _________
☐Unknown

§ *see Attachment I: Instructions for Investigator*

TREATMENT (CONTINUED)				
☐**Arginine**				
☐On-going	*specify effect of this treatment*:	☐No apparent	☐Positive effect	☐Unknown
☐Discontinued	*specify reason(s) for discontinuation*:	☐Lack of efficacy	☐Non-compliance	
		☐Lack of safety	☐ Other: _________	
☐Unknown				
☐**Carnitine**				
☐On-going	*specify effect of this treatment*:	☐No apparent	☐Positive effect	☐Unknown
☐Discontinued	*specify reason(s) for discontinuation*:	☐Lack of efficacy	☐Non-compliance	
		☐Lack of safety	☐ Other: _________	
☐Unknown				
☐**Other, specify** _____________________				
☐On-going	*specify effect of this treatment*:	☐No apparent	☐Positive effect	☐Unknown
☐Discontinued	*specify reason(s) for discontinuation*:	☐Lack of efficacy	☐Non-compliance	
		☐Lack of safety	☐ Other: _________	
☐Unknown				
☐**Other, specify** _____________________				
☐On-going	*specify effect of this treatment*:	☐No apparent	☐Positive effect	☐Unknown
☐Discontinued	*specify reason(s) for discontinuation*:	☐Lack of efficacy	☐Non-compliance	
		☐Lack of safety	☐ Other: _________	
☐Unknown				

ELECTRONIC SIGNATURE

I have carefully checked all data recorded in the electronic Case Report Form and I confirm that they are true, complete and accurate to the best of my knowledge.

Please enter your password:

Attachment I: Instructions for Investigator

Table INCLUSION/EXCLUSION CRITERIA - Inclusion criteria, page 1
**Inclusion criterion #2**: Neuroimaging findings include increased T2 signal on MRI or low attenuation on CT.

Table MEDICAL HISTORY - Perinatal History, page 1
**Perinatal History – Birth weight - IUGR**<br>AGA (appropriate for gestational age): between the 10th and 90th percentile for gestational age<br>SGA (small for gestational age): below the 10th percentile for gestational age<br>LGA (large for gestational age): above the 90th percentile for gestational age<br>IUGR (intra-uterine growth restricted): diminished growth velocity of the fetus on serial ultrasonographic scans.

Table MEDICAL HISTORY - Perinatal History, page 1
**Perinatal History – Head circumference at birth**<br>Normal: between –2SD and +2SD for gestational age<br>Microcephaly: below –2SD for gestational age<br>Macrocephaly: above +2SD for gestational age

Table MEDICAL HISTORY - Other dysfunction, page 3
**Failure to thrive:** weight for age that falls below the 5th percentile on multiple occasions or weight deceleration that crosses two or more major percentile lines on a growth chart over time

Table MEDICAL HISTORY - Other dysfunction, page 3
**Mental retardation:** categorization will be based on the DSM-IV and ICD 10 criteria for mental retardation. According to ICD 10:<br>Mild mental retardation: IQ range 50-69<br>Moderate mental retardation: IQ range 35-49<br>Severe mental retardation: IQ range 20-34<br>Profound mental retardation: IQ below 20

Table MEDICAL HISTORY - Other dysfunction, page 3
**Progressive microcephaly:** deceleration of head circumference that crosses two or more major percentile lines on a growth chart over time

Table DISEASE COURSE, page 4
**Disease course:** Acute exacerbation/relapse is defined as a worsening of patient's condition lasting longer than 24h, accompanied by significant deterioration of patient's motor function, requiring hospitalization and acute intervention.

Table SURVIVAL STATUS, page 4
**Survival status**<br>**Current status:** patient's status at the time of data registration on the present CRF<br>**Lost-to-follow-up**: specify last date of follow-up either in your site or elsewhere, if applicable

Table GROWTH, page 4
**Growth:** measurements at last visit. This also applies in case of death or lost-to-follow-up.<br>Example: weight for age -1.5 SD

Table LABORATORY FINDINGS, page 4
**Laboratory findings:** for lactate and pyruvate absolute values, please provide the maximum available values. For the lactate/pyruvate ratio, please provide the absolute value of lactate in CSF / the absolute value of pyruvate in CSF, taken at the same time. For the CSF/serum albumin ratio, the maximum available value will be provided.

Table MRI FINDINGS, page 7
**Date of 3rd MRI:** In case of more than three MRIs available, register data on the last MRI performed.
