# Supplementary material for: A multicenter study on Leigh syndrome: disease course and predictors of survival
Source: Orphanet J Rare Dis. 2014 Apr 15;9:52. doi: 10.1186/1750-1172-9-52 (PMC4021638; doi:10.1186/1750-1172-9-52)
Supplement: Additional file 2: Table S2 — Abnormal motor findings at disease onset versus later. [file 1750-1172-9-52-S2.doc]

**Additional file 2: Table S2: Abnormal motor findings at disease onset versus later**

| **Additional Table 2** | | | | | |
| --- | --- | --- | --- | --- | --- |
| **Motor sign** | **Total (n)*** | **Presentation at disease onset (n)** | **%** | **Presentation later (n)** | **%** |
| Hypotonia | 95 | 77 | 81 | 18 | 19 |
| Abnormal tendon reflexes | 61 | 19 | 31 | 42 | 69 |
| Dystonia | 57 | 15 | 26 | 42 | 74 |
| Babinski sign | 53 | 12 | 23 | 41 | 77 |
| Spasticity | 45 | 9 | 20 | 36 | 80 |
| Hypertonia | 42 | 14 | 33 | 28 | 67 |
| Ataxia | 36 | 16 | 44 | 20 | 56 |
| Muscle weakness | 34 | 14 | 41 | 20 | 59 |
| Other dyskinesia^ | 27 | 9 | 33 | 18 | 67 |
| Paresis/palsy | 26 | 11 | 42 | 15 | 58 |
| Chorea/athetosis | 25 | 5 | 20 | 20 | 80 |
| Cavus feet | 10 | 0 | 0 | 10 | 100 |
| Myoclonus | 9 | 0 | 0 | 9 | 100 |
| Hypokinesia/ bradykinesia | 5 | 2 | 40 | 3 | 60 |

*Patients with abnormal motor findings of unknown onset are not included in the present table

^ Tremor (n=7); dysarthria (n=4); dyscoordination (n=3); cogwheel phenomenon (n=3); stereotypies (n=3); opisthotonus (n=2); hyperkinesia (n=2); dyspraxia (n=1); jitteriness (n=1); sleeping through phenomenon (n=1)
