# Supplementary material for: A multicenter study on Leigh syndrome: disease course and predictors of survival
Source: Orphanet J Rare Dis. 2014 Apr 15;9:52. doi: 10.1186/1750-1172-9-52 (PMC4021638; doi:10.1186/1750-1172-9-52)

**Additional file 3: Figure 1** Predictors of survival

Additional Figure 1a: Kaplan-Meier survival curve by gender


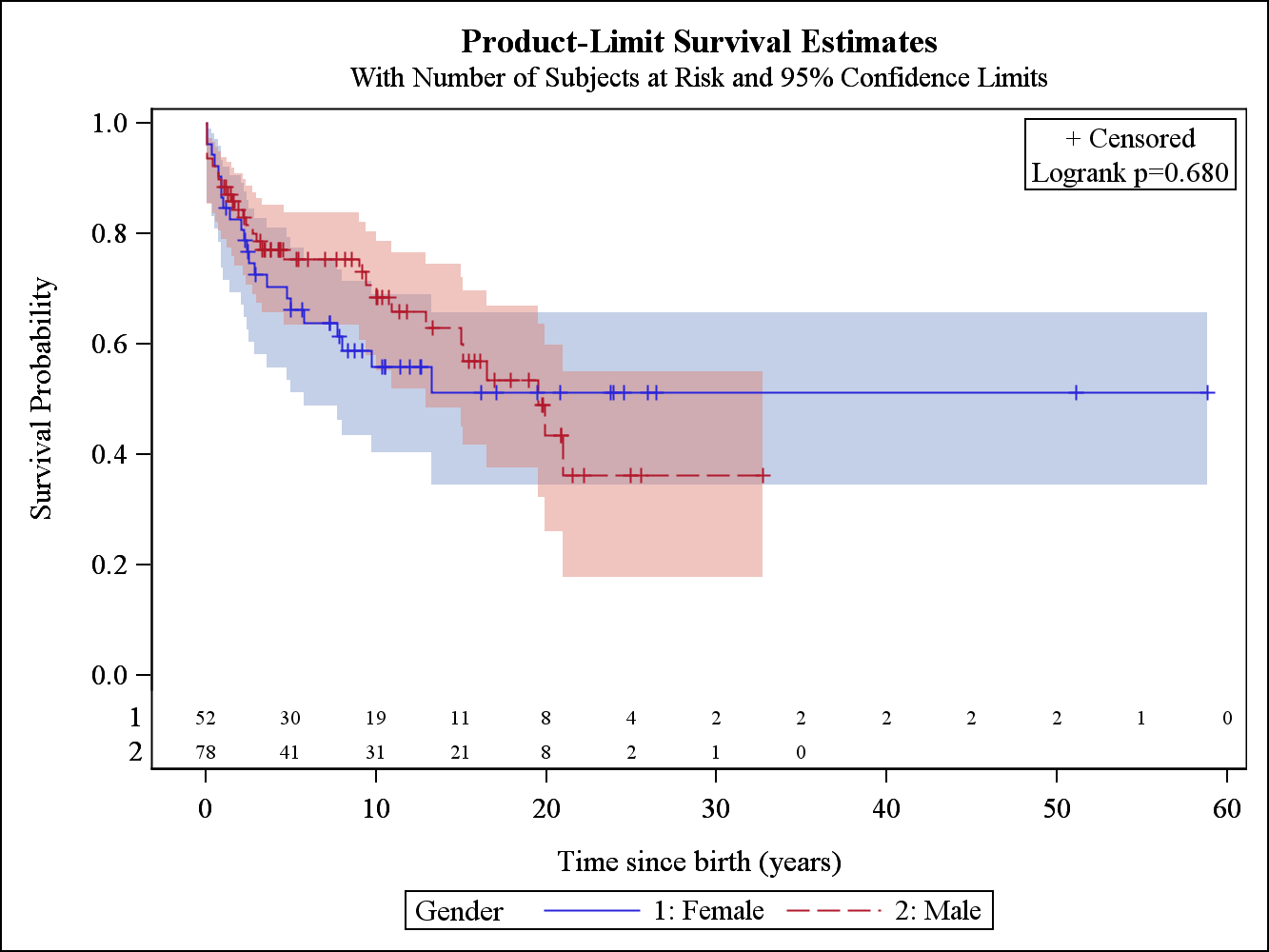


Additional Figure 1b: Kaplan-Meier survival curve for those patients with versus without pathological signs at birth


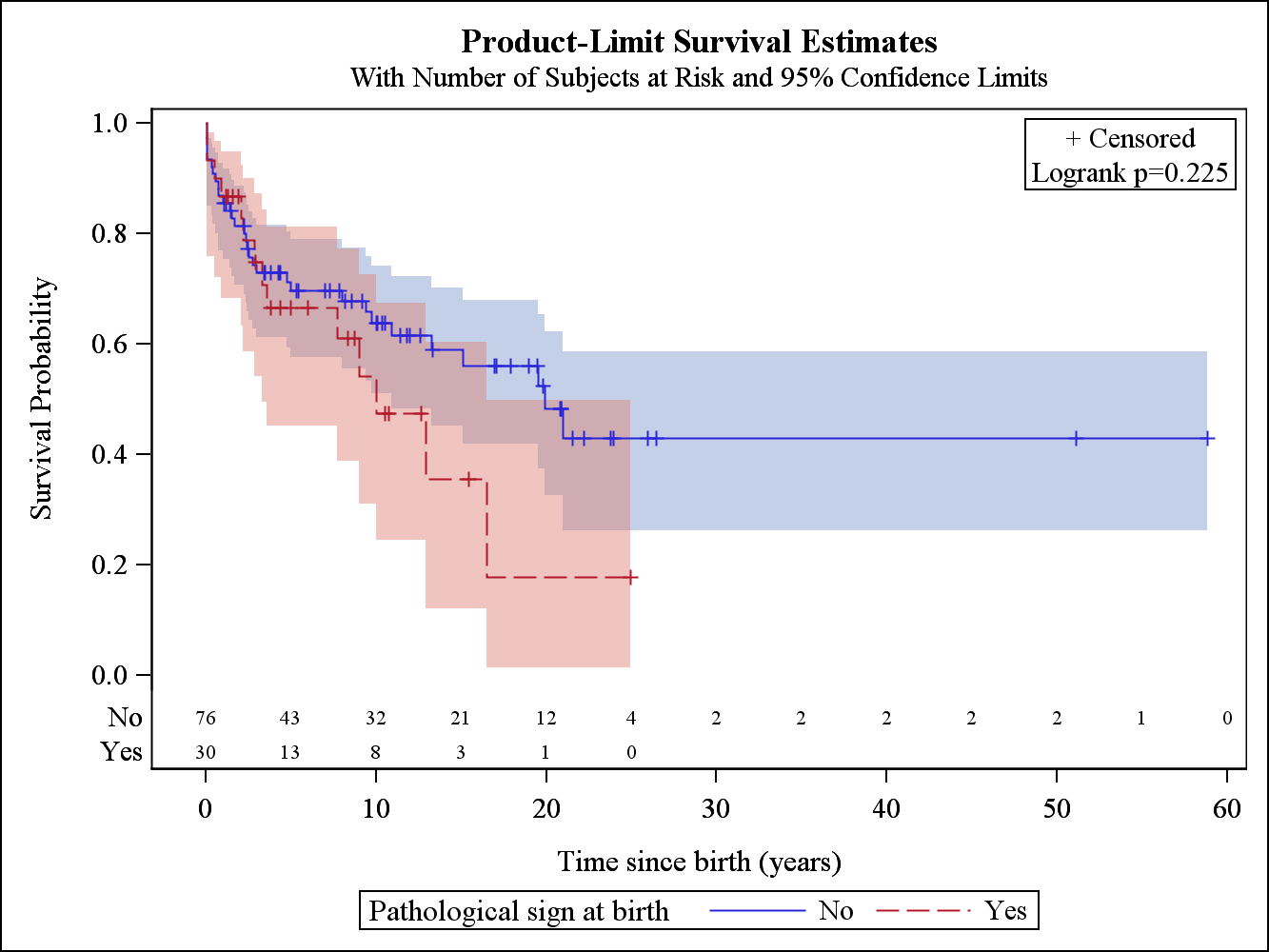


Additional Figure 1c: Kaplan-Meier survival curve for those patients with versus without cardiac dysfunction


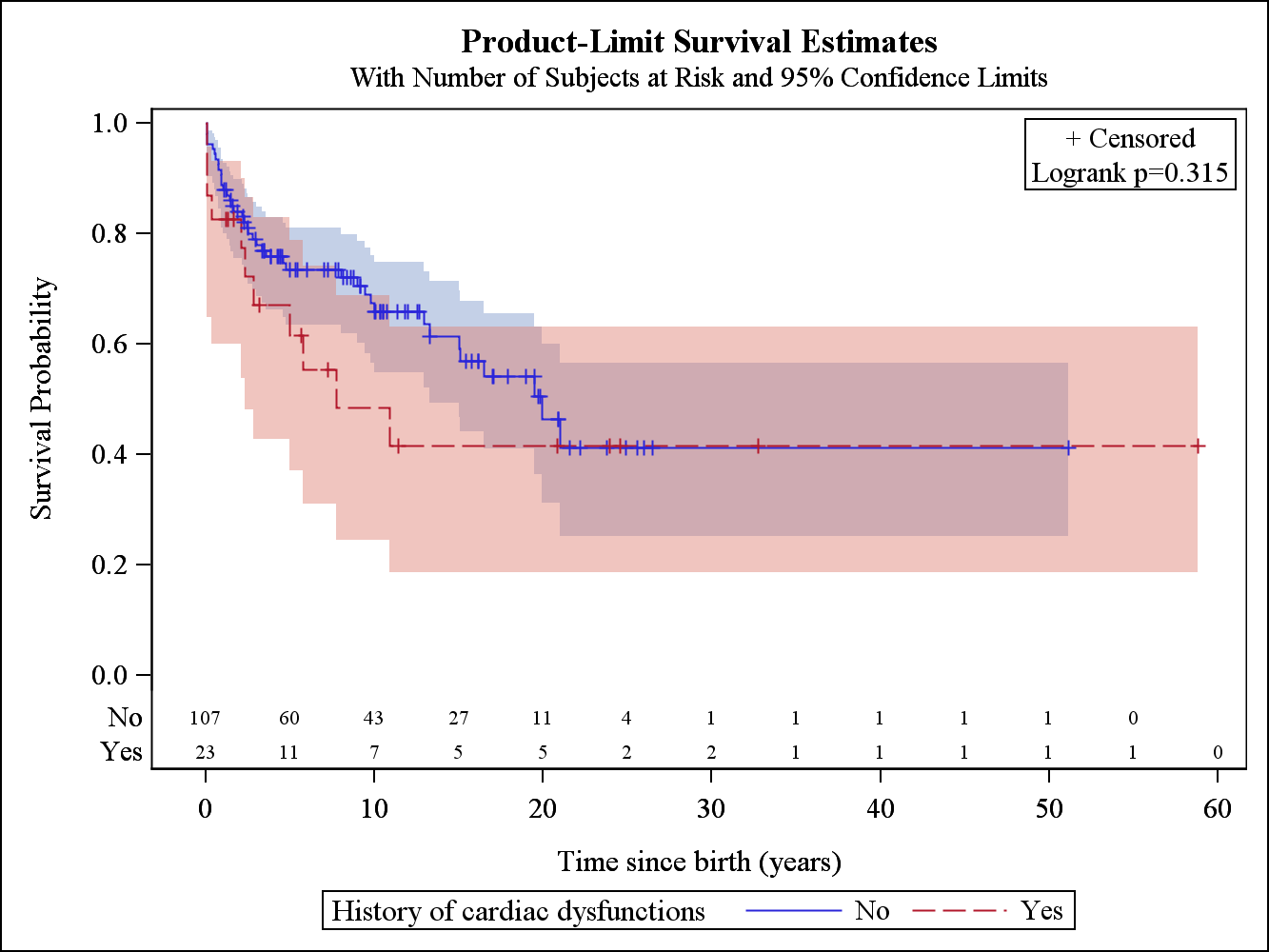


Additional Figure 1d: Kaplan-Meier survival curve for those patients with versus without hepatic dysfunction


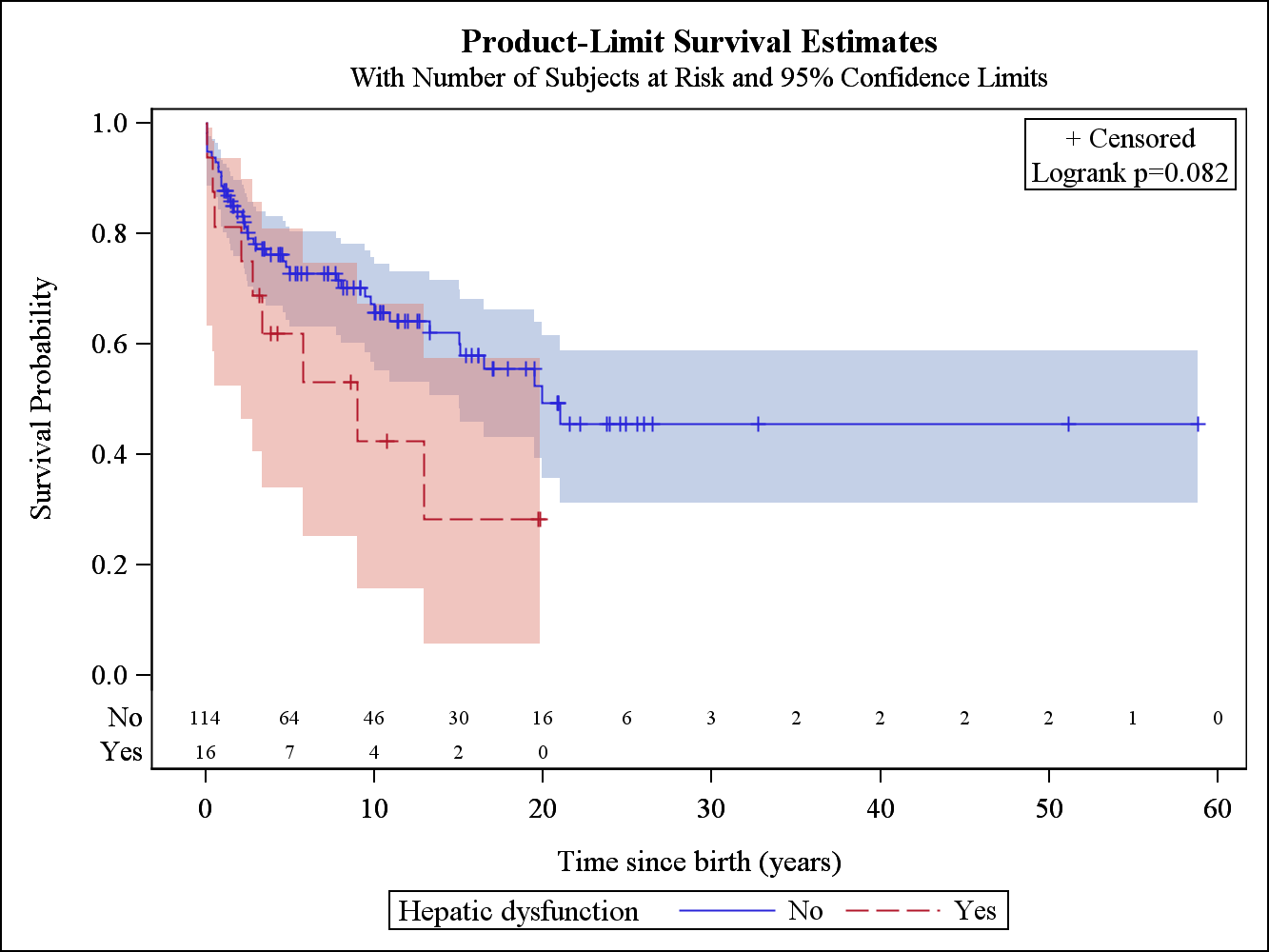


Additional Figure 1e: Kaplan-Meier survival curve by treatment with coenzyme Q10 in patients that survived at least 1 year


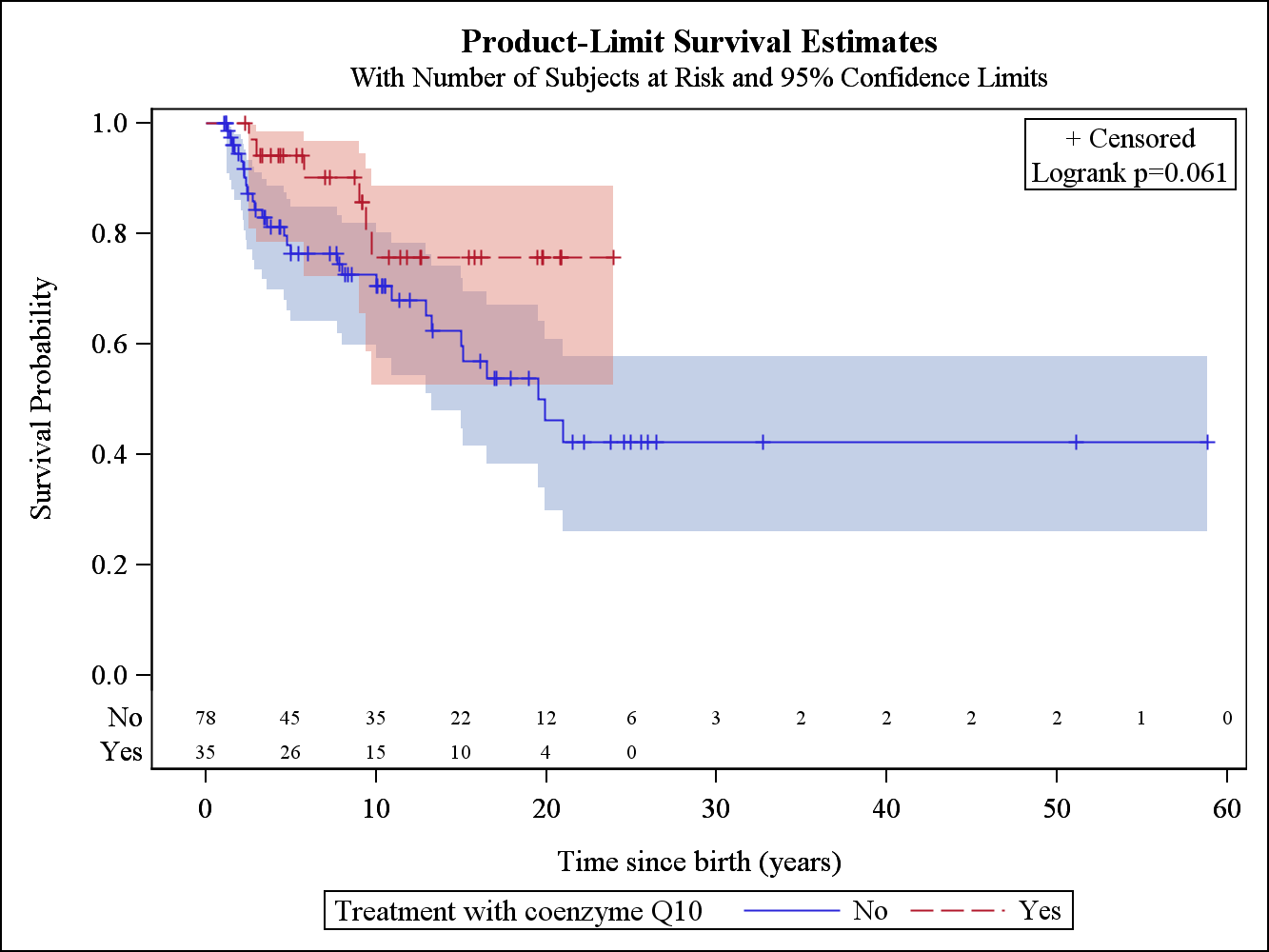

Supplement: Additional file 3: Figure S1 — Predictors of survival. [file 1750-1172-9-52-S3.doc]
